# Superior Control of HIV-1 Replication by CD8+ T Cells Targeting Conserved Epitopes: Implications for HIV Vaccine Design

**DOI:** 10.1371/journal.pone.0064405

**Published:** 2013-05-31

**Authors:** Pratima Kunwar, Natalie Hawkins, Warren L. Dinges, Yi Liu, Erin E. Gabriel, David A. Swan, Claire E. Stevens, Janine Maenza, Ann C. Collier, James I. Mullins, Tomer Hertz, Xuesong Yu, Helen Horton

**Affiliations:** 1 Viral Vaccine Program, Seattle Biomedical Research Institute, Seattle, Washington, United States of America; 2 Statistical Center for HIV Research and Prevention, Vaccine and Infectious Disease Division, Fred Hutchinson Cancer Research Center, Seattle, Washington, United States of America; 3 Polyclinic Infectious Disease, Seattle, Washington, United States of America; 4 Department of Medicine, University of Washington School of Medicine, Seattle, Washington, United States of America; 5 Department of Microbiology, University of Washington School of Medicine, Seattle, Washington, United States of America; 6 Department of Laboratory Medicine, University of Washington School of Medicine, Seattle, Washington, United States of America; 7 Department of Global Health, University of Washington School of Medicine, Seattle, Washington, United States of America; University of Alabama, United States of America

## Abstract

A successful HIV vaccine will likely induce both humoral and cell-mediated immunity, however, the enormous diversity of HIV has hampered the development of a vaccine that effectively elicits both arms of the adaptive immune response. To tackle the problem of viral diversity, T cell-based vaccine approaches have focused on two main strategies (i) increasing the breadth of vaccine-induced responses or (ii) increasing vaccine-induced responses targeting only conserved regions of the virus. The relative extent to which set-point viremia is impacted by epitope-conservation of CD8^+^ T cell responses elicited during early HIV-infection is unknown but has important implications for vaccine design. To address this question, we comprehensively mapped HIV-1 CD8^+^ T cell epitope-specificities in 23 ART-naïve individuals during early infection and computed their conservation score (CS) by three different methods (prevalence, entropy and conseq) on clade-B and group-M sequence alignments. The majority of CD8^+^ T cell responses were directed against variable epitopes (p<0.01). Interestingly, increasing breadth of CD8^+^ T cell responses specifically recognizing conserved epitopes was associated with lower set-point viremia (r = - 0.65, p = 0.009). Moreover, subjects possessing CD8^+^ T cells recognizing at least one conserved epitope had 1.4 log_10_ lower set-point viremia compared to those recognizing only variable epitopes (p = 0.021). The association between viral control and the breadth of conserved CD8^+^ T cell responses may be influenced by the method of CS definition and sequences used to determine conservation levels. Strikingly, targeting variable versus conserved epitopes was independent of HLA type (p = 0.215). The associations with viral control were independent of functional avidity of CD8^+^ T cell responses elicited during early infection. Taken together, these data suggest that the next-generation of T-cell based HIV-1 vaccines should focus on strategies that can elicit CD8^+^ T cell responses to multiple conserved epitopes of HIV-1.

## Introduction

An efficacious prophylactic HIV-1 vaccine will likely need to elicit both HIV-1-specific antibodies and T cell responses, as there is evidence that both arms of the adaptive immune system play an important role in viral control (reviewed in refs.[1]–[3]). Most previous candidate HIV-1 vaccines designed to induce protective antibody or CD8^+^ T cell responses have failed to prevent infection or reduce viral load (reviewed in ref [4]). The recent RV144 trial has only shown a marginal protection in preventing infection without an effect on viral load [5] and this modest protection appears to be mediated by antibody responses [6]. However, the immunogens included in the RV144 vaccine may not be optimal for eliciting protective T cell responses. Indeed the most effective prophylactic vaccines tested to date in non-human primates (NHP) have all induced robust CD8^+^ T cell responses that correlate with protection [7], [8]. These studies underscore the necessity to optimize immunogens to induce both humoral and cell-mediated arms of the adaptive immune system.

Several lines of evidence demonstrate the role of CD8^+^ T responses in controlling or preventing HIV infection providing a strong rationale for renewed efforts to optimize T cell-based immunogens [3], [9]. Evidence for control of established infection is emphasized by studies showing the link between HLA types and viral control [10]–[12]. Although the majority of infected people progress to AIDS within 10 years without antiretroviral therapy, the pace of clinical progression is highly variable. Certain MHC class I alleles are associated with rapid (B*35Px: B*35∶02, B*35∶03, B*35∶04, and B*53∶01) vs. slow (B*27, B*57) progression to AIDS [13], [14] implicating a role for CD8^+^ T cells in HIV control. In addition, the extensive literature on viral escape from CD8^+^ T cells [15]–[17] supports the view that this arm of immune system is applying pressure on the virus. While it has been repeatedly shown that T cells can control established infection, there is also convincing evidence that CD8^+^ T cells can prevent infection. The presence of cross-reactive HIV-specific CD8^+^ T cell responses in highly exposed but persistently uninfected women [18]–[21] suggest that CD8^+^ T cells can prevent HIV infection. Previous studies have shown that a live attenuated SIV vaccine (SIVmac239Δnef) confers a significant level of protection against heterologous SIV challenge [22], [23]. CD8^+^ T cells depletion studies further demonstrated that this protection was indeed mediated by cellular immune responses [22]. Moreover, other studies by this group have shown that induction of SIV-specific effector memory T cells using a CMV vector provides protection against SIV infection [8]. While data on HIV control and prevention do support efforts to design T cell based HIV vaccines, the extraordinary worldwide diversity of HIV presents a huge challenge. To tackle this problem, T cell-based vaccine approaches have come up with two main strategies: the Mosaic Immunogen, which emphasizes increasing the breadth of vaccine-induced responses [24], [25], and conserved immunogens, which emphasize increasing the breadth of vaccine-induced T-cell responses only to highly conserved viral regions [26]–[29]. While both approaches are theoretically sound, there are no current data suggesting that either approach will be successful in inducing T cells with superior antiviral efficacy.

Despite a considerable research effort in this area, the qualities that define “protective” HIV-specific CD8^+^ T cells are still unknown, making rationale design of vaccines difficult. While numerous studies provide strong evidence that CD8^+^ T cells play an important role in immune control of HIV, a significant number of virus-specific CD8^+^ T cells are also detectable in individuals who fail to control viremia [30], [31], suggesting that not all CD8^+^ T cells responses are created equally but that they differ significantly in their ability to control viral replication. Although the mechanism(s) underlying these differences are not entirely clear, understanding them is critical for development of HIV vaccines. Four qualities of HIV-1-specific CD8^+^ T cells have been previously suggested to play an important role in controlling HIV-1 replication: frequency, breadth, functionality and specificity. HLA-tetramer based studies initially demonstrated an inverse correlation between frequency of A*0201-restricted HIV-specific CD8^+^ T cells and plasma viral load [32]. However, subsequent IFN-γ ELISPOT-based studies examining the frequency of IFN-γ-secreting HIV-specific CD8^+^ T cells did not show an inverse correlation with viral load [31], [33]–[36]. Similarly, the breadth of HIV-specific CD8^+^ T cell responses has not shown an inverse correlation with plasma viral load [33], [34]. Increased poly-functionality and proliferative capacity of CD8^+^ T cells has been associated with slow HIV disease progression [37]–[40], although a recent study showed no association between antiviral efficacy and poly-functionality, or proliferative capacity of CD8^+^ T cells [41]. Thus, to date, the precise qualities of effective epitope-specific CD8^+^ T cell responses that may be responsible for immune control of HIV remain unclear. Multiple studies have shown an inverse correlation between Gag-specific CD8^+^ T cell responses and viral load [42], [43], and an association between CD8^+^ T cell responses targeting conserved regions with improved disease outcome [44], [45]. Studies showing a positive correlation between escape in the well-defined B*27-restricted KK10 epitope and increased viral load [16], [46] suggest that CD8^+^ T cells recognizing certain epitopes are efficient at controlling viral replication. In addition, CD8^+^ T cell responses restricted by favorable alleles have been shown to target highly conserved regions (Gag p24) of the virus and escape from these Gag-specific CD8^+^ T cell responses was either not possible or occurred with a coincident viral fitness cost [47], [48]. Furthermore, we have shown that the association between HIV-1 disease progression and distinct MHC class I alleles is linked to CD8^+^ T cells recognition of conserved HIV-1 epitopes early in infection [49]. Although, these studies suggest that the epitope-conservation of CD8^+^ T cells plays an important role in mediating control of HIV infection, a comprehensive study that examines the epitope-conservation of the complete CD8^+^ T cell repertoire in acutely infected therapy naïve individuals, and how conservation score (CS) of the recognized epitopes correlates with viral control has not been conducted to date.

In the present study, we comprehensively mapped the CD8^+^ T cell response in 23 therapy naïve individuals during early HIV-1 infection, to address the question of whether CS of CD8^+^ T cell epitopes plays an important role in viral control. Additionally, we assessed the impact of breadth, magnitude and functional avidity of CD8^+^ T cell responses elicited in early HIV-1 infection in viral control. Our data provide the first clear evidence that the majority of CD8^+^ T cell responses elicited during early HIV-1 infection in therapy naïve individuals are directed against variable epitopes. The data show that the breadth of initial CD8^+^ T cell responses recognizing conserved HIV epitopes is important for the subsequent control of viremia. Furthermore, we show that individuals possessing CD8^+^ T cells directed against at least one conserved epitope early in infection have lower viral load (VL) set point than those individuals possessing CD8^+^ T cells directed against only variable epitopes. These findings suggest that broad HIV-specific CD8^+^ T cell responses specifically recognizing conserved epitopes elicited during early infection are superior at controlling viral replication *in vivo*, providing important implications for rational design of future T cell-based immunogens.

## Materials and Methods

### Ethics Statement

The Institutional Review Boards at the University of Washington and Seattle BioMedical Research Institute approved the study. All adults provided written informed consent.

### Study Subjects

HIV-specific CD8^+^ T cell responses were characterized in 23 HIV-1-infected therapy naïve subjects from Seattle, Washington. These subjects were selected from the University of Washington Primary Infection Cohort (PIC) based on availability of specimens from ART naïve subjects within 6 months post infection (except one 7.5 months post infection). The mean days-post-infection (dpi) was 84 (range 16–226 days). The PIC cohort's estimated HIV-1 infection date was used as the date of infection; this was the symptom onset date for symptomatic subjects or the midpoint between the last negative and first positive HIV tests in those lacking symptoms. Before July 2003, blood HIV-1 viral-load testing employed a branched-chain DNA (bDNA) assay (Chiron Diagnostics), which had a lower limit of detection of 500 HIV-1 RNA copies per mL [50]. After 2002, an in-house real-time reverse transcription (RT)-PCR method was used, with a lower limit of detection of 50 copies per ml [51]. Most of these subjects were Caucasian men who have sex with men (MSM). Clinical information and HLA genotype of these subjects is summarized in Table 1 and Table S1 respectively. Longitudinal clinical data were collected from all study participants. VL set point was defined as the mean of available viral load results from 91–426 days (3–15 months) dpi prior use of ART (Table 1). Of the 23 subjects, 15 subjects had at least one VL set point result (mean 5.3, range 1–13 values) during this period.

**Table 1:** Assessment of VL set point of subjects with primary HIV-1 infection.

PTID	Race	Est. HIV date	Sample aDPI	SetpointVL	bSPVL#	SPVL DPI Range
11439	Caucasian	10/6/05	35	17,424	6	91–375
53617	Caucasian	6/2/01	177	340	8	107–415
21746	Caucasian	5/26/01	150	46	13	124–417
71101	Caucasian	5/19/04	16	136,253	5	96–321
75688	Caucasian	8/18/04	44	5,554	4	202–426
41325	Caucasian	10/13/05	34	59,540	5	109–333
94153	Caucasian	12/30/05	96	1,604	9	96–396
20786	Caucasian	7/9/06	22	48,069	6	94–346
17543	Mixed/Other	1/25/07	36	21,525	1	151
25122	Caucasian	4/20/07	47	225,585	3	139–243
53653	Caucasian	6/23/07	65	77,654	4	108–352
51314	Mixed/Other	6/4/08	226	1,314	4	103–217
44149	Caucasian	4/7/10	177	965	5	128–350
10849	Mixed/Other	7/25/10	108	6,011	5	108–365
67200	Caucasian	9/24/10	194	1,712	2	173–194
25327	Caucasian	1/14/09	69	none		
63794	Caucasian	2/23/09	16	none		
79379	Caucasian	7/23/09	83	none		
57604	Mixed/Other	7/28/09	79	none		
98621	Caucasian	8/22/09	67	none		
32645	Caucasian	2/10/10	69	none		
51729	Mixed/Other	3/15/10	44	none		
44091	Caucasian	5/22/10	81	none		

a DPI, days post infection.

b SPVL#, number of longitudinal viral load data used to identify viral load set point.

### Epitope Mapping of T cell Response (IFN-γ ELISPOT)

Cryopreserved peripheral blood mononuclear cells (PBMC) were thawed and rested overnight at 37°C before plating 100,000–200,000 PBMC per well in IFN-γ ELISPOT assays (Millipore), as previously described [40], [52]. Briefly, PBMC were stimulated with master pools of up to 100 peptides. These peptides were 15-mers overlapping by 11 amino acids spanning the entire coding sequence of HIV-1 (Potential T Cell Epitope [PTE] peptide sets, provided by NIH AIDS Research & Reagent Program). Once a positive response to a master pool was observed, PBMC were stimulated with sub pools in a matrix system of 8–15 15mers per pool to determine the individual 15mer giving the T cell response [53], [54]. These 15mers were then tested individually to confirm recognition. Unstimulated cells served as a negative control, and phytohemagglutinin (PHA; Remel) stimulated cells served as a positive control. Biosyn Corp., and Sigma-Aldrich synthesized all HLA class I restricted peptides (8- to 11-mer). Peptides were used at a final concentration of 2 µg/mL. The number of spot forming cells (SFC) was calculated by subtracting the mean number of spots in the negative control wells from the mean number of spots for each stimulation condition. An IFN-γ result was considered positive when the background-subtracted number of SFC was twice the background (negative control) and at least 50 SFC per million PBMC.

MHC class I-restricted CD8^+^ T cell responses were further mapped. For each of the 15mers mediating MHC class I-restricted CD8^+^ T cell response, optimal epitopes were predicted based on an individual’s HLA type from LANL DB and tested for reactivity by IFN-γ ELISPOT. If no known epitopes were predicted, overlapping 9-mers encompassing the 15-mer were synthesized and tested for reactivity by IFN-γ ELISPOT to identify the optimal epitope sequence. Epitopes were defined as novel epitopes (i) if the epitope had not been previously defined in the LANL DB and (ii) if the subject did not possess the known restricting allele of the previously defined epitopes.

### Class I MHC Restriction

IFN-γ ELISPOT assay was used to determine MHC restriction of each of the epitopes with no known HLA restriction as previously described [33]. Briefly, a panel of Epstein-Barr Virus (EBV)-transformed B-cell lines (BLCL) was mismatched with the initial responder except for one HLA allele. Each BLCL was incubated with or without the epitope (2 µg/mL) at 37°C for 3 hours, washed four times in phosphate-buffered saline (PBS), and incubated with reactive PBMC for 20 hours. The remainder of the ELISPOT assay was performed as described above.

### TCR Functional Avidity

We used a previously described method [33], with modifications, to identify functional avidities of epitope-specific CD8^+^ T cells using standard IFN-γ ELISPOT with 5-fold serial dilutions of their cognate epitopes ranging from 100 µg/mL to 5.12×10^−6^ µg/mL. The molar peptide concentration was calculated based on the molecular weight of each peptide and plotted as a peptide concentration. The SFC per million of PBMC were plotted against the log_10_ peptide concentration. The peptide concentration (nM) that resulted in 50% of the maximum response (50% effective concentration [EC50]) was calculated with Prism software (version 5.0d; GraphPad Software).

### Definition of Conserved Versus Variable Epitopes

Conservation scores (CS) were computed using three different approaches which have been previously used in the HIV field: (1) Epitope prevalence scores [55], [56], (2) Shannon entropy [57] and (3) conseq [58], [59]. All of these scores use a set of aligned HIV sequences as input. In this work, we considered both a clade-B and a group-M alignments [29] downloaded from the Los Alamos HIV-1 sequence database (LANL DB) of 2005, and all scores were computed using the same alignments. Unique clade-B and group-M amino acid sequences were downloaded from the LANL DB (http://www.hiv.lanl.gov). To avoid potential bias due to the submission of partial sequences to the LANL DB, we only used complete or near-complete sequences in the analysis. For clade-B: 197 (Env), 198 (Gag), 97 (Pol), 327 (Vif), 225 (Vpr), 203 (Vpu), 243 (Nef), 299 (Tat), 175 (Rev) and for group-M: 871 (Env), 619 (Gag), 615 (Pol), 967 (Vif), 835 (Vpr), 925 (Vpu), 1224 (Nef), 1178 (Tat), 938 (Rev) unique sequences were used to calculate the CS. By using both clade-B and group-M alignments, we obtained conservation estimates based on sequences that were clade matched to our clinical cohort (clade-B) as well as those based on more diverse set of sequences from all clades (group-M).

#### Epitope prevalence scores

This score is based on the frequency of a given epitope in a set of circulating clade-B (bCSp) or group-M (mCSp) sequence alignments. Epitopes were classified into two groups as described previously with a slight modification [56]: *Conserved* epitopes were defined as those that were ≥80% prevalent in a given sequence set, and *relatively conserved* epitopes were ones that occurred between 50–80% in the data set. All epitopes with prevalence scores ≤50% were considered *variable*.

#### Shannon entropy scores

Entropy is a common measure for conservation that measures the level of uncertainty in a random variable [57]. This measure is computed independently for each position in an aligned set of circulating clade-B (bCSe) or group-M (mCSe) sequences. Positions with low entropy are more conserved than those with high entropy. The entropy CS of an epitope is the average entropy scores of all positions along the epitope [60]–[62]. In order to define thresholds for conserved vs. variable epitopes using this score, we used the distribution of 9 mer scores over all HIV proteins. Conserved epitopes were defined as epitopes with entropy CS ≤20^th^ percentile of this distribution (i.e., were more conserved than 80% of all potential 9 mers). Different cutoffs were computed for entropy CS computed on the clade-B or clade-M alignments.

#### Conseq

Is based on building a maximum parsimony tree to calculate a conservation score for each site by considering the number of substitutions at each site weighted by their physicochemical distance [58], [59], [63]. As for entropy scores, each site in the target protein has an associated conseq score, and lower scores indicate higher conservation. The conseq CS for a given epitope is the average conseq scores of all positions along the epitope. In order to determine a conservation threshold for conseq, we calculated the scores for all 9 mers in all HIV proteins and combined to make a global distribution of 9 mer scores. Conserved epitopes were those with conseq CS of ≤20^th^ percentile of this empirical distribution. We created separate distributions and cutoffs for the clade-B and group-M alignments.

### Statistical Analysis

Subjects’ VL set points were log_10_ transformed for analysis to create a more symmetrical distribution. The distributions of a continuous variable, such as VL set point, were compared between two groups using a Mann Whitney (MW) test or paired data in two groups using a Wilcoxon matched-pair signed rank (WSR) test, among three or more groups using a Kruskal-Wallis Rank Sum (KW) test or paired data among three or more groups using a Friedman test. The differences in mean magnitude of CD8^+^ T cell responses by protein types, estimated by a generalized estimating equation, was compared using a Wald test. Correlations between two continuous variables, such as impact of breadth or magnitude of CD8^+^ T cell responses on VL set point, were computed using the Spearman Rank Correlation, denoted r. The association between two categorical variables was assessed using a Fisher’s exact test. A univariate linear model was used to assess the effect of each variable, such as conserved response, on VL set point. A multivariate model was used to examine the relationship between CS or breadth or interaction effect of CS and breadth on VL set point. P values were not adjusted for multiple comparisons. P values less than 0.05 were considered significant. All analyses were performed using Prism software (version 5.0d) and R statistical software (version 2.13.0).

## Results

### Assessment of HIV-1-specific T-cell Responses Using a Potential T Cell Epitope [PTE] Peptide Set-based IFN-γ ELISPOT Assay

Twenty-three HIV-infected antiretroviral therapy (ART)-naïve subjects from Seattle were evaluated for epitope specificity, breadth, magnitude and functional avidity of HIV-1-specific CD8^+^ T cell responses elicited during early infection. The median time to evaluation was 69 days post infection. To increase the probability of identifying responses to novel epitopes as well as increase the ability to accurately assess the breadth of T cell responses, we used PTE peptide sets [64] to map the T cell responses against the entire HIV-1 proteome. The breadth and magnitude of virus-specific T-cell responses differed significantly among individuals at different stages of HIV-1 infection (Table S1). We found broader CD8^+^ T cell responses in people who have been infected for a longer period of time (Spearman Rank Correlation, r = 0.63, p<0.002, Figure S1). The median number of CD8^+^ T cell epitopes detected was 7 (range, 3–13), the mean magnitude of the epitope-specific T cell response was 779 SFC/10^6^ PBMC (median, 516; range, 57–3836) and the mean total magnitude was 5838 SFC/10^6^ PBMC (median, 5069; range 970–14906). The HIV-1-specific CD8^+^ T cells induced during early infection in the 23 individuals recognized a total of 123 distinct optimal epitopes (8–11mers) spanning all HIV-1 proteins except Vpu and were restricted by 27 distinct class I alleles (Table S1). Responses were detected to Gag in 19 (83%), Pol in 17 (74%), Env in 17 (74%), Nef in 15 (65%) and other auxiliary proteins in 11 (48%) of 23 subjects. Interestingly, 39 (32%) epitopes were novel (Table S1). The MHC restriction of 28% of these novel epitopes was identified. These novel epitopes were discovered in Env (n = 13), Pol (n = 11), Gag (n = 8), auxiliary proteins (n = 6) and Nef (n = 1). Taken together, in agreement with previous findings [65], these data indicate that the use of only known HLA restricted epitopes would lead to an under-estimation of CD8^+^ T cell response in HIV-1-infected individuals.

### Early CD8^+^ T cell Responses are Preferentially Directed against Variable Epitopes

**Figure 1:**
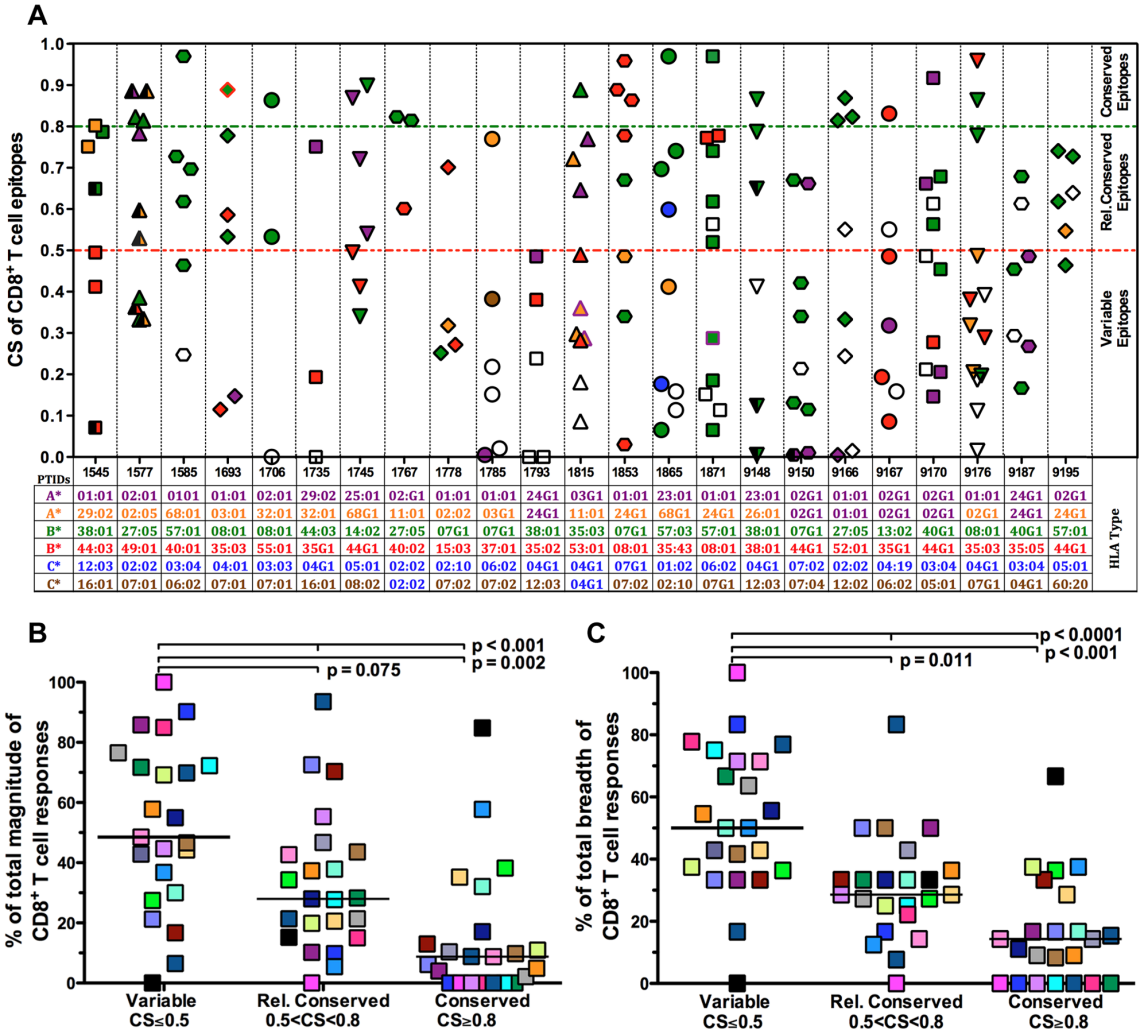
Variable epitopes are preferentially recognized in early HIV-1-infection. A total of 123 epitopes identified in 23 subjects with early HIV-1-infection were analyzed according to their CS. (A) Epitopes were plotted against their CS. The HLA genotype of each subject is also shown. CS of each epitope was calculated as the frequency of an exact epitope match in aligned clade-B sequences (bCSp). Each symbol represents an epitope recognized by HIV-specific CD8^+^ T cells in an individual. Symbols are colored to denote the HLA restriction of the epitope. (B) The percentage of total magnitude of IFN-γ responses directed against epitopes by CS grouping (Friedman, p<0.001). (C) The percentage of total breadth of CD8^+^ T cell responses directed against epitopes by CS grouping (Friedman, p<0.0001). (B–C) The % of the total magnitude and breadth of CD8^+^ T cell responses targeting “Variable”, “Relatively Conserved” and “Conserved” epitopes. Individual subjects are denoted by a specific color. Horizontal lines indicate median values; statistical significance was assessed by matched test for 2 groups (Wilcoxon matched-pairs signed rank test) or multiple groups (Friedman test).

The conservation score (CS) of a targeted epitope was defined as the proportion of HIV-1 clade-B amino acid sequences (bCSp) in the LANL DB that include the exact epitope. *Conserved* and *variable* epitopes were defined as those that were prevalent in ≥80% and ≤50% in a given sequence alignment, respectively. The majority of epitopes targeted early in HIV infection 60% (73 out of 123), were variable, and only 13% (16 out of 123) were conserved (Figure 1A). Most of the conserved responses were directed to Gag and Pol epitopes. The median CS of targeted Gag and Pol epitopes was higher than the median CS of Env epitopes (MW, p = 0.033, and p = 0.001, respectively, Figure S2A). However, the magnitude of responses elicited by Gag and Pol epitopes was not significantly different than those elicited by Nef, Env and Acc epitopes (Wald, p = 0.930, Figure S2B). Twenty-five percent (4/16) of conserved epitopes identified in our study were restricted by favorable alleles. Three of these conserved epitopes KRWIILGLNK (B*27-KK10), KRKGGIGGY (B*27-KY9) and KAFSPEVIPMF (B*57-KF11) were recognized in 100% of individuals who possessed these alleles. In contrast, only one conserved epitope (TVLDVGDAY) was restricted by an unfavorable allele (B*35∶01). The remaining 69% (11/16) of conserved epitopes identified in our study were restricted by neutral alleles (Table S1). Most of the novel CD8^+^ T cell epitopes, 74% (29/39) were variable and only 5% (2/39) were conserved. Furthermore, we found that in any individual subject, significantly higher proportions of CD8^+^ T cell responses (in both magnitude and breadth) were directed against variable epitopes than those directed against conserved epitopes (WSR, p = 0.002, Figure 1B and p<0.001, Figure 1C respectively). A median of 49% of the total magnitude and 50% of the total breadth of CD8^+^ T cell responses were directed against variable epitopes. Collectively, this emphasizes the fact that most of the early CD8^+^ T cell responses to HIV-1 are directed against variable epitopes.

### Lower Viral Load is Associated with CD8^+^ T cell Responses Directed to Conserved Epitopes

**Figure 2:**
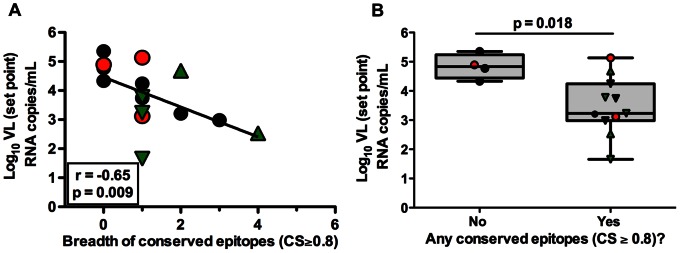
CD8^+^ T cell responses against conserved epitopes (bCSp) are associated with viral control. (A) The plasma VL set point was compared to breadth of conserved epitopes (Spearman Rank Correlation, r = −0.65, p = 0.009). The solid line represents a regression line. (B) The median plasma viral set point in individuals who mounted CD8^+^ T cell responses against at least one conserved epitope (Mann Whitney, p = 0.018). (A–B) Subjects possessing B*35Px, B*27 and B*57 alleles are represented by red circles, green triangles and inverted green triangles respectively.

To evaluate the impact of CD8^+^ T cells targeting conserved epitopes on the control of viral replication, we compared the breadth of CD8^+^ T cell responses targeting only conserved epitopes with plasma VL set point. Interestingly, increasing breadth of CD8^+^ T cell responses recognizing conserved epitopes was associated with lower viremia (r = –0.65, p = 0.009, Figure 2A). Next, we compared the plasma VL set point of individuals who mounted responses against at least one conserved epitope to individuals who did not mount such a response. Individuals possessing CD8^+^ T cell recognizing even one conserved epitope had lower VL set point than those who did not recognize any conserved epitope (MW, p = 0.018, Figure 2B). In a univariate linear model, the mean VL set point in subjects who mounted CD8^+^ T cell responses against at least one conserved epitope was 1.4 log_10_ lower than those who failed to target at least one conserved epitope (95% CI: −2.5, −0.24, p = 0.021). Collectively, these data suggest that possessing CD8^+^ T cells recognizing conserved epitopes is associated with lower VL set point.

### Targeting Conserved Epitopes was Independent of Possession of Favorable Alleles

**Figure 3:**
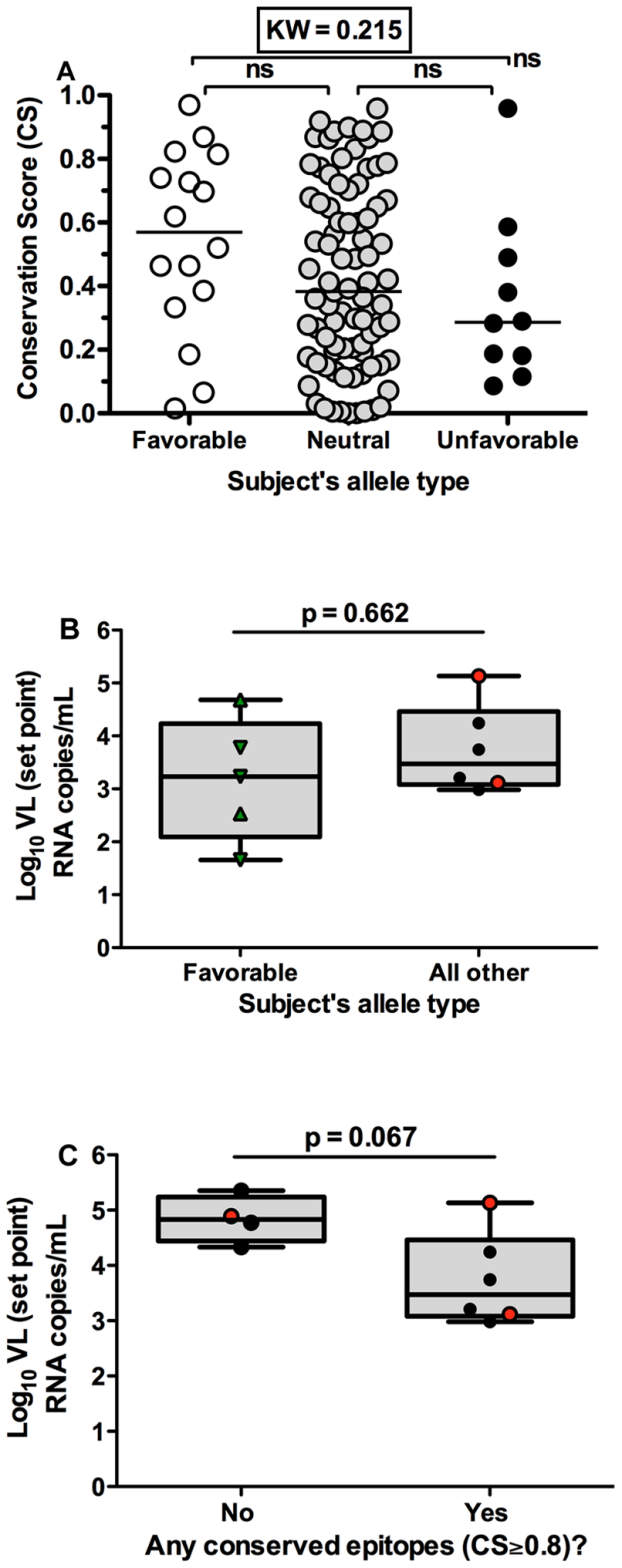
Targeting conserved epitope (bCSp) was independent of possession of favorable alleles. (A) The median CS of epitopes by HLA group: favorable alleles (subjects possessing B*27 and B*57 alleles), unfavorable alleles (subjects possessing B*35Px alleles: B*35Px: B*35∶02, B*35∶03, B*35∶04, and B*53∶01) and neutral alleles (subjects not possessing B*27, B*57 or B*35Px alleles) (Kruskal-Wallis, p = 0.215). Horizontal lines indicate median value. (B) The median plasma VL set point in individuals (subjects not recognizing at least one conserved epitopes were excluded on this analysis) recognizing at least one conserved epitope by possession of favorable allele (Mann Whitney, p = 0.662). (C) The median plasma VL set point in individuals (not possessing favorable alleles, subjects possessing favorable alleles were excluded) who elicited CD8^+^ T cell responses against at least one conserved epitope (Mann Whitney, p = 0.067). (B–C) Subjects possessing B*35Px, B*27 and B*57 alleles are represented by red circles, green triangles and inverted green triangles respectively.

Given that five of the eleven individuals who mounted CD8^+^ T cell responses against conserved epitopes had a favorable HLA allele, we next assessed whether the association between CD8^+^ T cells recognizing conserved epitopes and lower VL set point may have been due to the fact that these conserved epitopes were recognized by CD8^+^ T cells restricted by favorable alleles. CS of CD8^+^ T cells epitopes restricted by favorable alleles (such as HLA-B*27, HLA-B*57), neutral alleles and unfavorable alleles (such as HLA-B*35Px alleles: B*35∶02, B*35∶03, B*35∶04, and B*53∶01) was compared. Surprisingly, the median CS of CD8^+^ T cell epitopes restricted by favorable alleles was not significantly different from those restricted by neutral or unfavorable alleles (KW, p = 0.215, Figure 3A).

To assess whether the association between CD8^+^ T cells recognizing conserved epitopes with lower VL set point may have been due to the possession of favorable alleles, we compared average plasma VL set point in individuals with their HLA types. Unexpectedly, the median plasma VL set points were not significantly different by HLA grouping although there was a trend for lower VL set point in individuals possessing favorable alleles (KW, p = 0.296, Figure S3 A–C). Next, we performed a subset analysis excluding all the subjects who did not recognize any conserved epitopes in order to assess the impact of favorable alleles on VL set point. There was no statistically significant difference in VL set points between individuals with or without favorable alleles (MW, p = 0.662, Figure 3B). In another subset analysis, we excluded subjects possessing favorable alleles in order to specifically compare VL set points in subjects who recognized at least one conserved epitope versus those who did not recognize any conserved epitopes. Although, we did not have enough power to detect differences, there was a trend of lower VL set point in subjects who mounted CD8^+^ T cell responses against at least one conserved epitope (MW, p = 0.067, Figure 3C). Overall, these data suggest that possessing CD8^+^ T cells recognizing conserved epitopes regardless of the MHC restriction is associated with lower VL set point.

### Breadth of CD8^+^ T cell Responses to Conserved Gag Epitopes is Associated with Lower VL Set Point

We next investigated the contribution of breadth of CD8^+^ T cell responses to the control of viral replication. The total breadth of CD8^+^ T cell responses elicited during early HIV-1 infection was significantly inversely correlated with the VL set point (r = − 0.55, p = 0.035, Figure S4A). In order to avoid a bias introduced from subjects mounting CD8^+^ T cell responses against conserved epitopes, we performed further subset analysis excluding subjects who mounted responses against conserved epitopes. Interestingly, in the absence of individuals mounting responses against conserved epitope, the breadth of CD8^+^ T cell responses no longer remained significantly inversely correlated with the VL set point (Spearman Rank Correlation, r = 0.80, p = 0.333, data not shown). We also used a multivariate model to examine the joint effects of CS and breadth of CD8^+^ T cell responses on plasma VL set point and found that the main effects of both CS and breadth and interaction between them are not significant (data not shown). Similar to the subset analysis above, VL set point is lowered when targeting conserved epitopes in addition to increasing breadth.

We further assessed the relationship between the breadth of CD8^+^ T cell responses elicited by Gag epitopes and plasma VL set point. The breadth of Gag and Pol epitopes targeted during early HIV-1 infection was correlated inversely with VL set point (r = − 0.64, p = 0.010, and r = −0.69, p = 0.005, Figure S4B and S4C respectively). In contrast, there was no significant correlation between breadth of Nef-specific, Env-specific and other Auxiliary protein-specific CD8^+^ T cell response with VL set point (data not shown).

**Figure 4:**
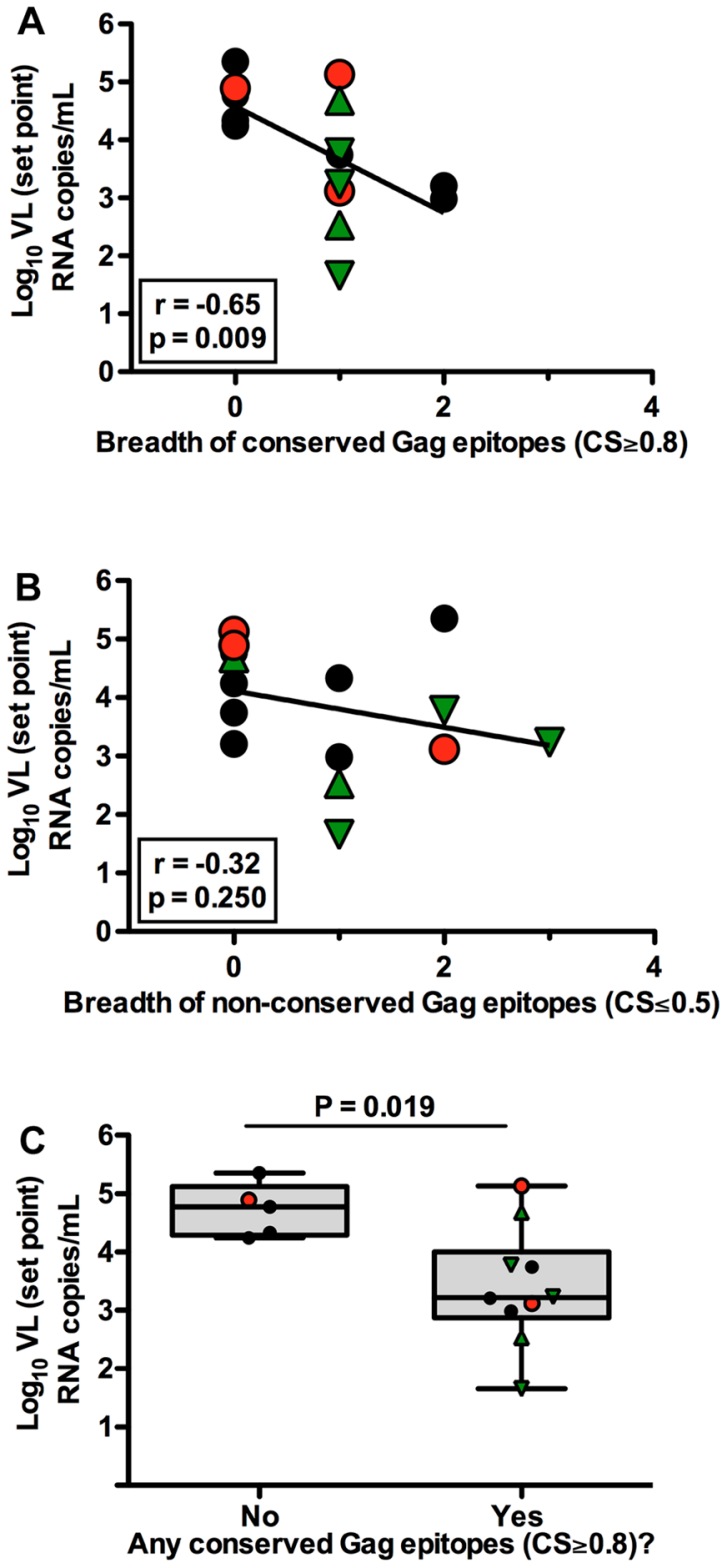
Breadth of HIV-1-specific CD8^+^ T cell responses to conserved Gag epitopes correlate with lower viremia. VL set point was compared to breadth of CD8^+^ T cell responses. (A and B) Correlation between breadth of CD8^+^ T cell responses against conserved Gag or variable Gag epitopes (clade-B) with plasma VL set point (Spearman Rank Correlation, r = −0.65, p = 0.009 and r = −0.32, p = 0.250 respectively). (A–B) The solid line represents a regression line. (C) The median plasma VL set point in individuals who mounted CD8^+^ T cell responses against at least one conserved (bCSp) Gag epitope (Mann Whitney, p = 0.019). (A–C) Subject possessing B*35Px, B*27 and B*57 allele are represented by red circles, green triangles and inverted green triangles respectively.

We next compared breadth of conserved Gag epitopes or conserved Pol epitopes with VL set point in order to identify the contribution of CD8^+^ T cell responses recognizing conserved Gag or Pol epitopes in viral control. We found a statistically significant inverse correlation between breadth of conserved Gag epitopes and VL set point (r = −0.65, p = 0.009, Figure 4A). In contrast, the breadth of conserved Pol epitopes did not significantly correlate with VL set point (r = −0.33, p = 0.226, data not shown). Strikingly, the breadth of CD8^+^ T cell responses to variable Gag epitopes did not significantly inversely correlate with the VL set point (Spearman Rank Correlation, r = 0.32, p = 0.250, Figure 4B). We subsequently compared the VL set point in subjects who mounted responses against at least one conserved Gag or Pol epitope compared to subjects who did not mount such responses. Interestingly, there was lower VL set point in individuals who mounted at least one conserved Gag response versus those who did not (MW, p = 0.019, Figure 4C). In contrast, subjects mounting CD8^+^ T cell responses against at least one conserved Pol epitope did not have significantly lower VL set point than those who did not mount such responses (p = 0.279, data not shown). The estimated difference between subjects mounting responses to at least one conserved Gag epitope and those who did not was −1.0 log_10_ (95% CI: −2.1, 0.15; p = 0.082). Overall, these data suggest that the previous associations between breadth of Gag responses and lower viral load may have been driven exclusively by only the conserved Gag epitopes that were targeted.

### The Association between CD8^+^ T cell Responses Directed against Conserved Epitopes and Viremia is Independent of Functional Avidity

**Figure 5:**
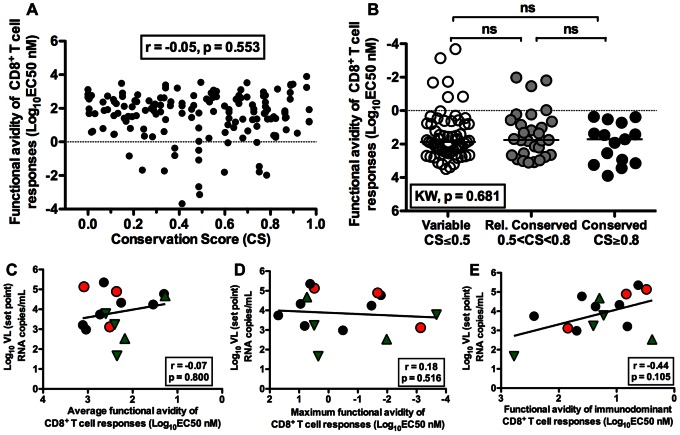
CD8^+^ T cell functional avidity and control of viral replication. (A) Correlation between functional avidity and CS of CD8^+^ T cell epitopes. (B) The functional avidity of CD8^+^ T cells by CS (clade-B) grouping (Kruskal-Wallis, p = 0.681). Horizontal lines indicate median values. (C–E) Correlation between average (C), maximum (D) and immunodominant (E) functional avidity of CD8^+^ T cells in each subject with average plasma viral set point (Spearman Rank Correlation, r = −0.07, p = 0.800; r = −0.18, p = 0.516 and r = −0.44, p = 0.105 respectively). (C–E) The solid line represents a regression line. Subject possessing B*35Px, B*27 and B*57 allele are represented by red circles, green triangles and inverted green triangles respectively.

We next assessed whether CD8^+^ T cell responses with high functional avidity have an impact on the VL set point. For this purpose, we assessed the log_10_-transformed EC_50_ of all CD8^+^ T cell responses. There was no correlation between functional avidity of CD8^+^ T cell responses and the CS of T cell epitopes (r = −0.05, p = 0.553, Figure 5A). We then compared the functional avidities of CD8^+^ T cell responses elicited by epitopes that were variable, relatively conserved and conserved. The median functional avidities of CD8^+^ T cell responses were not different by CS grouping (KW, p = 0.681, Figure 5B). Interestingly, there was no significant difference between median functional avidities of CD8^+^ T cell responses elicited by variable epitopes compared to those elicited by conserved epitopes. To test whether high functional avidities of CD8^+^ T cell responses play an important role in viral control, we compared the functional avidities of average, maximum and immunodominant epitope-specific CD8^+^ T cell responses with plasma VL set point. In line with the above findings, we found no significant correlation between functional avidities of average, maximum or immunodominant CD8^+^ T cell responses with VL set point (r = −0.07, p = 0.800, Figure 5C; r = 0.18, p = 0.516, Figure 5D; and r = −0.44, p = 0.105 Figure 5E, respectively). Taken together, these data suggest that the association between viral control and CD8^+^ T cells recognizing conserved epitopes is not due to the possibility that these CD8^+^ T cells might have higher functional avidities.

### Method of Determining CS Influences Significance of T cell Association with Viral Control

Since we observed a significant inverse association of viral control and CD8^+^ T cells targeting conserved epitopes based on clade-B alignments, we further wanted to investigate if the same pattern exists if epitope-conservation of CD8^+^ T cell epitopes were defined using different sequence alignments with different methods. For this purpose, the CS of CD8^+^ T cell epitopes was defined based on clade-B or more diverse group-M alignments with two different methods, (i) clade-B prevalence CS (bCSp) or group-M prevalence CS (mCSp): the proportion of clade-B (as defined in Figure 2) or group-M amino acid sequences in the LANL DB that include the epitope, and (ii) clade-B entropy CS (bCSe) or group-M entropy CS (mCSe): defined based on the Shannon entropy score calculated for each position in all circulating clade-B or group-M alignments. While the cutoff for conserved epitopes based on prevalence scores is ≥80%, the cutoff for conserved epitopes based on entropy scores or conseq scores is ≤20%. The CS of epitopes defined by different methods is shown in Table S2.

**Figure 6:**
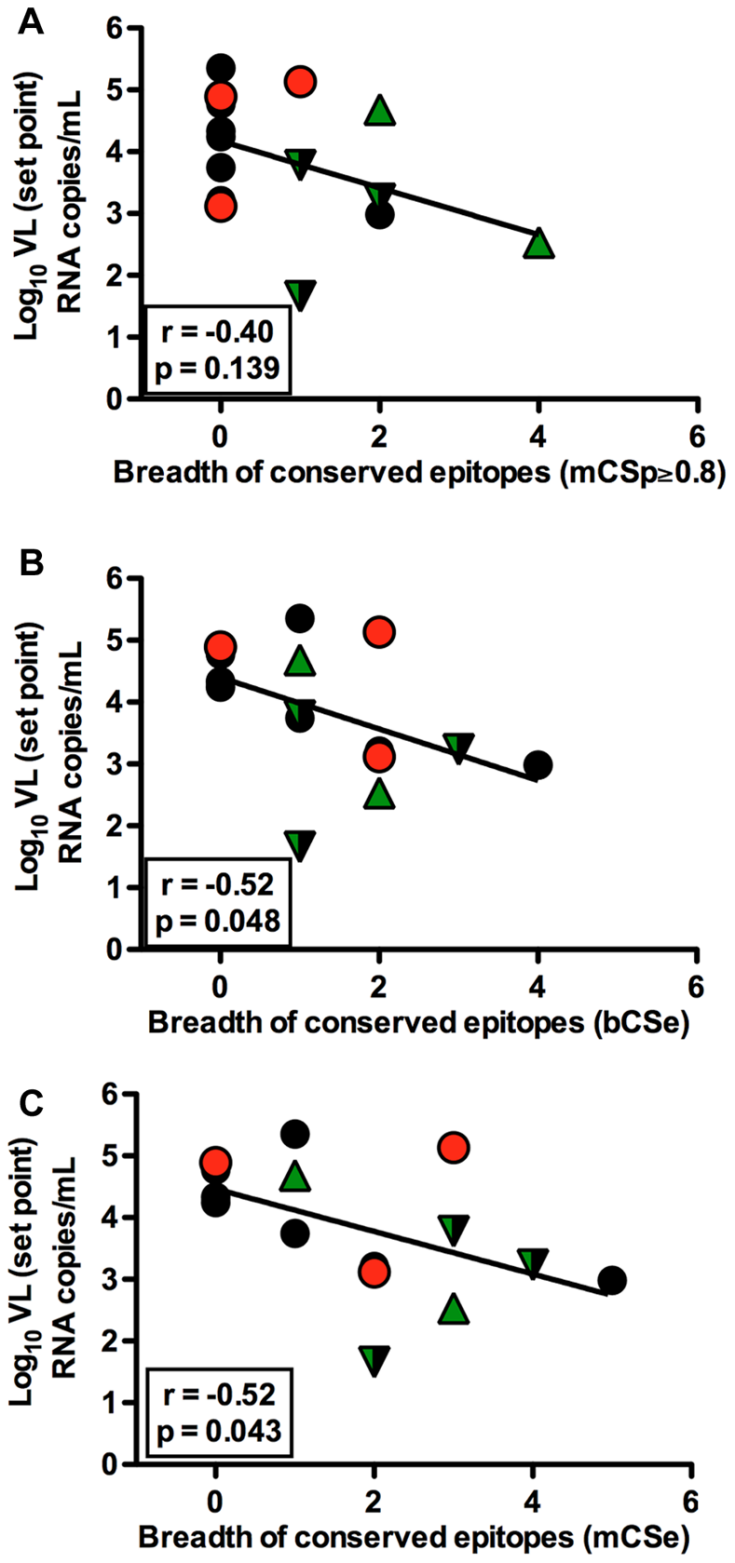
Method of determining CS influences significance of T cell association with and viral control. (A) The plasma VL set point was compared to breadth of conserved epitopes based on mCSp (Spearman Rank Correlation, r = −0.40, p = 0.139). (B, C) The plasma VL set point was compared to breadth of conserved epitopes based on bCSe (Spearman Rank Correlation, r = −0.52, p = 0.048) and mCSe (Spearman Rank Correlation, r = −0.52, p = 0.043) respectively. Subject possessing B*35Px, B*27 and B*57 allele are represented by red circles, green triangles and inverted green triangles respectively.

We compared the breadth of CD8^+^ T cells recognizing conserved epitopes using the different methods with plasma VL set point. Interestingly, the inverse correlation observed for bCSp did not reach statistical significance (2A) when epitope-conservation was defined as mCSp (r = −0.40, p = 0.139, Figure 6A), although the data showed a trend in the same direction as was observed for the bCSp analysis. Remarkably, the breadth of CD8^+^ T cells recognizing conserved epitopes based on entropy inversely correlated with VL set point whether entropy was defined based on clade-B or group-M alignments (r = −0.52, p = 0.048, Figure 6B and r = −0.52, p = 0.043, Figure 6C, respectively). We next assessed whether the association between CD8^+^ T cells targeting conserved epitopes and lower VL set point may have been due to the fact that these conserved epitopes were recognized by CD8^+^ T cells restricted by favorable alleles. For this, CS of CD8^+^ T cells epitopes restricted by favorable alleles (e.g. HLA-B*27, HLA-B*57), neutral alleles and unfavorable alleles (e.g. HLA-B*35Px alleles) was compared. The median CS of CD8^+^ T cell epitopes restricted by favorable alleles was not significantly different from those restricted by neutral or unfavorable alleles regardless of method of definition CS or type of alignment used to define CS (KW, p = 0.215, Figure 3A for bCSp; KW, p = 0.394 for mCSp, data not shown; KW, p = 0.283 for bCSe, data not shown, and; KW, p = 0.215 for mCSe, data not shown). Overall, these data suggest that epitope-conservation of CD8^+^ T cell response play an important role in viral control and that the sequences used to compute CS may impact the level of association for some methods.

## Discussion

Understanding the characteristics that define the correlates of protection against HIV-1 infection would guide rational design of effective vaccines. Although CD8^+^ T cell responses elicited during early HIV-1 infection are thought to be important in containment of HIV-1 (reviewed in ref. [3], [15], [66]), the qualities that define effective versus ineffective CD8^+^ T cells are still not known. To date, studies correlating early HIV-1-specific CD8^+^ T cell responses and viral control have been restricted to the breadth and magnitude of IFN-γ secreting HIV-1-specific CD8^+^ T cells [33], [34], [67], [68]. Thus, relative contributions of epitope-conservation, as related to CS of the overall epitope-specific CD8^+^ T cell responses elicited during early HIV-1 infection and their relationship to HIV control, are largely unclear. Furthermore, most studies investigating the impact of breadth of CD8^+^ T cell responses elicited during early HIV-1 infection in viral control have focused on samples from treated individuals during early HIV-1-infection [34], [67] and a panel of HLA-restricted epitopes [67], [68] or a limited selection of HIV-1 proteins [67] rather than using samples from therapy naïve individuals and peptide sets spanning the whole HIV-1 proteome. As far as we are aware, there is only one published study analyzing the comprehensive CD8^+^ T cell responses elicited during early HIV-1 infection in therapy naïve individuals using overlapping peptide sets spanning the HIV-1 proteome based on clade-B consensus sequences [33]. However, this study did not look at the relative contribution of specificity or CS of the recognized epitopes on viremia control. We therefore investigated the relative contribution of breadth compared with epitope-conservation of CD8^+^ T cell responses elicited during early HIV-1 infection in viral control. As a secondary objective, we also assessed magnitude, functional avidity and MHC utilized to mount HIV-1-specific CD8^+^ T cell responses in early HIV-1 infection in viral control. For this, we performed a comprehensive mapping of CD8^+^ T cell responses directed towards all HIV-1 proteins in 23 therapy naïve subjects using PTE peptide sets. We examined the relationship between the qualities of early CD8^+^ T cell responses and VL set point. To the best of our knowledge, no studies have determined whether conserved or variable epitopes are preferentially targeted during early in HIV-1-infection comprehensively and how this is related to subsequent viral control.

PTE peptides are 15mers that are designed to encompass all potential 10-mer epitopes that are present at 15% or greater in current circulating group-M strains [64]. These peptide reagents have been shown to increase the number of responses detected in infected individuals over consensus B reagents [69]. Data from other investigators have demonstrated that use of autologous peptide sets enhances the ability to detect responses in HIV-1-infected individuals over consensus clade-B peptide sequences [65], [70]. It is possible that the use of autologous peptide sets would have detected more responses against variable epitopes than PTE peptide sets, however, since our study already shows that the majority of responses elicited during early infection target variable epitopes, it is not clear that any additional insight would have been gained using autologous peptide sets, which would only contain additional variable epitopes. Although PTE peptides are not autologous peptides, they still contain multiple variants of each potential epitope, and should enhance the probability of detecting all the T cell responses elicited within an individual. Indeed, 60% (74/123) of epitopes recognized during early HIV infection were variable, 24% (29/123) of epitopes identified were novel epitopes and 74% (29/39) of those had a CS ≤0.5 (based on clade-B). Similarly, when CS were computed based on more diverse, group-M sequences, 76% (94/123) of epitopes targeted early in HIV infection were variable, 28% (35/123) of epitopes identified were novel epitopes and 90% (35/39) of those had a CS of ≤0.5. These results suggest that the use of only known HLA restricted epitopes would underestimate the true number of targeted CD8^+^ T cell epitopes in HIV-1-infeciton. Because of the comprehensive analysis of CD8^+^ T cell responses with PTE peptides in patients with diverse HLA types, it is not surprising that so many new epitopes in variable regions were identified.

In our study, the median number of CD8^+^ T cell responses elicited during early HIV-1 infection was 7 (range, 3–13), which is higher than previous studies have reported (median, 2–4; range, 0–7) [33], [34], [67]. The observed differences could be at least partially attributable to human genetic variation, sampling time after infection, treatment status [34], [67], and use of a more comprehensive peptide set since the previous studies used peptides based on clade-B consensus sequences [33]. Our results demonstrate that the majority of CD8^+^ T cell responses are directed against variable epitopes, which is not surprising since HIV-1 has such a highly variable genome, and therefore, by chance, the frequency of CD8^+^ T cell response against conserved epitopes would be lower. However, this contrasts with findings made in a previous study showing that both conserved and variable epitopes are recognized with a similar probability by CD8^+^ T cells [55]. The observed differences could be due to differences in the definition of conserved epitopes and the peptide sets (containing predefined optimal epitopes) used to map CD8^+^ T cell responses. Our finding is in agreement with a study [56] that looked at whether the T cell responses elicited by the Merck Ad5 vaccine (containing HIV-1 Gag, Pol and Nef) were directed against conserved or variable epitopes. Furthermore, this finding is also in line with a previous study showing that CD8^+^ T cell responses in early infection are directed towards high entropy 15–20 mer peptides (i.e., lower amino acid conservation) compared to chronic infection [60]. However, they only studied specificity of CD8^+^ T cell responses at the 15 mer peptide level based on entropy alone, and they did not look at whether CD8^+^ T cell responses mounted against lower entropy epitopes were associated with viral control.

We attempted to define conservation of recognized epitopes by two different methods. We found a significantly lower VL set point in subjects possessing CD8^+^ T cells targeting conserved epitopes compared to those not targeting conserved epitopes when epitope-conservation was defined based on clade-B prevalence (bCSp), however, this association was less significant when the epitope-conservation was defined based on group-M prevalence (mCSp). Interestingly, the inverse association between the number of conserved epitopes with VL set point persists whether epitope-conservation was defined based on entropy in clade-B or the group-M (bCSe or mCSe). Collectively, these data suggest that CD8^+^ T cell responses targeting conserved epitopes of HIV would confer viral suppression, as mutation at these regions are likely to have a fitness cost for the virus, so they cannot easily escape. Although certain MHC class I alleles, notably B*27 and B*57, are associated with slow HIV disease progression [14], the relative contribution of the restricting allele versus the restricted epitope is not entirely clear. In this study, we found that CD8^+^ T cells restricted by favorable alleles do not always mount responses against conserved epitopes. This finding is in agreement with a recent study [71] that found that mounting CD8^+^ T cell responses against conserved elements is effective even if these responses are not restricted by favorable alleles. Although our study was limited by sample size, the lack of significant association between particular HLA type and viral control was consistent with previous finding by Kiepiela *et al*. [42], who did not see this association in chronic HIV-1-infection either. The previously well-defined relationship between HLA type and viral control may be different in different individuals as demonstrated in a previous study [72] which showed that HIV-1 adapts to CD8^+^ T cell responses restricted by alleles that are known to provide protection. However, our finding of lack of association between favorable alleles and viral control is surprising and contrasts with findings made in previous studies [12], [14], where there is a strong association between favorable alleles and HIV control during chronic infection in LTNPs, and more recently one mechanism of this HIV control has been elucidated [73]. This association may be less relevant during early HIV-1-infection, however, these observed differences could also be attributable to human genetic variation, as well as to the smaller sample size in our study. The lack of association between HLA type and viral control during early HIV infection does not rule out the well-defined relationship established during chronic infection. Overall, this finding may suggest that the correlation between CD8^+^ T cell responses targeting conserved epitope with viral control is potentially not due to the likelihood that these responses are always restricted by favorable alleles.

In contrast to a previous report [33], our study demonstrated that breadth and magnitude (data not shown) of CD8^+^ T cell responses in early HIV-1 infection inversely correlated with plasma VL set point. The reasons that our study demonstrated an association between CD8^+^ T cell responses and VL set point could be due to a number of factors. Firstly, in this study, we used PTE peptide sets, which would lead to a more comprehensive detection of CD8^+^ T cell responses. Secondly, we used plasma VL set point, not the concurrent VL, from each subject to avoid the chances of correlating with peak VL as we studied CD8^+^ T cell responses in early HIV-1 infection. This finding is in agreement with a study [67] that reported an inverse correlation between the breadth of CD8^+^ T cell responses and VL at the time of presentation in individuals who were treated before HIV-1 seroconversion. In agreement with previous studies, we found that the breadth of overall Gag-specific CD8^+^ T cell responses [42], [74] and magnitude (data not shown) of Gag-specific CD8^+^ T cell responses [74] contributes to viral control. However, these studies looked at association only in chronic HIV infection, and did not look at the relative contribution of conserved Gag versus non-conserved Gag responses in viral control. Our data showed that only conserved Gag-specific responses were correlated with control of viral load, not variable Gag-specific responses. Taken together, these findings support the hypothesis that early and strong CD8^+^ T cell recognition of conserved Gag-epitopes during the initial phase of HIV-1-infection is an effective contributor of viral control.

Several studies have demonstrated the potential importance of functional avidity of CD8^+^ T cells in HIV control [71], [75], [76]. In contrast, we did not see any correlation between functional avidity of CD8^+^ T cells and viral control. The observed differences could be attributable to the methodology to determine the functional avidity of CD8^+^ T cells (EC50 versus SD50) [71], [75] or in the methodology to calculate VL (plasma VL set point versus cell associated VL) [76]. Perhaps more importantly, the previous studies assessed CD8^+^ T cell responses in chronic infection, whereas our study assessed CD8^+^ T cell responses in early HIV-1-infection, and there has been a study showing that functional avidities change over time during infection [77]. Our finding indicates that there is no correlation between CS of CD8^+^ T cell epitopes and functional avidity of CD8^+^ T cells. Taken together, these findings suggest that functional avidity of CD8^+^ T cells may not be a crucial feature of immune control, at least during early HIV-1-infection.

A few caveats of our study must be considered. It is possible that the contribution of overall CD8^+^ T cell responses in viral control cannot be elucidated completely by the cross-sectional nature of our study. Second, our calculation of conservation scores equally weighted all positions in the epitope sequence based on clade-B or group-M sequences. Extending these analyses with CS of CD8^+^ T cell epitopes calculated by different approaches may further inform the effects of epitope-conservation in viral control. In this study, we also performed limited analysis with a third method for defining evolutionary conservation – Conseq [58], [59]. Interestingly, we did not find any associations between the Conseq conservation scores of CD8^+^ T cell responses and viral control. Another approach weighting amino acids based on their known interactions with major histocompatibility complex (MHC) and T cell receptor (TCR) could provide different results as mutations in these amino acids alter peptide binding to MHC and T cell recognition. Third, the definition of conserved epitope (conserved if CS≥0.8) was arbitrary since the B*27 restricted KRWIILGLNK epitope (which has been shown to be associated with viral control [40]) has a CS of 0.82 and 0.87 (based on clade-B and group-M alignments, respectively). Finally, due to the limited number of subjects who reached VL set point, our study lacks power to investigate the interaction between breadth, CS and HLA allele types. Larger studies will be needed to address these issues, although this may be difficult due to the current treatment guidelines of early initiation of therapy.

In summary, we find that breadth and epitope-conservation of HIV-specific CD8^+^ T cells elicited during early infection are important for controlling viral replication *in vivo.* From our limited sample size, it appears that these associations may be independent of possession of particular HLA types. From a vaccine design perspective, our study has profound implications. Given limited feasibility to design vaccines based on an individual’s HLA haplotype, our data suggest that a T cell based vaccine should be designed using conserved regions of HIV [29], regardless of the vaccine recipient’s HLA types. Additionally, we find that early CD8^+^ T cell responses elicited in the majority of individuals are preferentially directed against variable epitopes of the virus. Therefore designing an immunogen that excludes variable regions of HIV should counteract this pattern and would be advantageous to the host as the host has only the option of mounting immune responses against conserved regions of HIV. Such a vaccine approach will increase the likelihood that vaccine-induced CD8^+^ T cells will recognize incoming viral species of diverse clades and decrease the likelihood of rapid escape variants against the recognized epitopes. Overall, our results provide information that may guide the selection of immunogens for development of effective HIV-1 vaccines. Rational design of immunization approaches should aim at induction of greater breadth and magnitude of CD8^+^ T cell responses precisely recognizing only conserved epitopes of HIV.

## Supporting Information

Figure S1 **Assessment of HIV-1-specific CD8^+^ T cell responses during early HIV-1 infection.** The number of HIV-1-specific CD8^+^ T cell epitopes was plotted for each subject against the sampling time, shown as days post infection (dpi). Subjects possessing B*35Px, B*27 and B*57 alleles are represented by red circles, green triangles and inverted green triangles respectively.
(TIFF)

Figure S2 **Majority of conserved epitopes (bCSp) targeted in early HIV-1 infections are Gag and Pol.** The CS and magnitude of CD8^+^ T cell responses of total of 123 epitopes identified in 23 subjects were analyzed based on their protein types. (A) The median CS of CD8^+^ T cell epitopes by HIV protein types (Kruskal-Wallis, p = 0.022). (B) The median magnitude of responses (SFC/M) by protein types (Wald/GEE, p = 0.93). Horizontal lines indicate median.
(TIFF)

Figure S3 **HIV-1 VL set points are not significantly different by HLA types.** (A–C) The median plasma VL set point in individuals possessing favorable, unfavorable or all other alleles (Kruskal-Wallis, p = 0.296). Horizontal lines indicate median. Subjects possessing B*35Px, B*27 and B*57 alleles are represented by red circles, green triangles and inverted green triangles respectively
(TIFF)

Figure S4 **Correlation between breadth of HIV-1-specific CD8^+^ T cell responses and viremia.** (A) Correlation between total breadth of CD8^+^ T cell responses and average plasma VL set point (Spearman Rank Correlation, r = −0.55, p = 0.035). (B and C) Correlation between breadth of CD8^+^ T cell responses against Gag or Pol epitopes with plasma VL set point (Spearman Rank Correlation, r = −0.64, p = 0.010 and r = −0.69, p = 0.005 respectively). (A–C) The solid line represents a regression line. Subject possessing B*35Px, B*27 and B*57 allele are represented by red circles, green triangles and inverted green triangles respectively.
(TIFF)

Table S1 **HIV-1 specific CD8^+^ T cell responses in early infection: epitope specificity, MHC restriction, and frequency.**
(DOCX)

Table S2 **HIV-1 specific CD8^+^ T cell responses in early infection: comparison of CD8^+^ T cell epitope-conservation by different methods.**
(DOCX)
