# Supplementary figures and images for: Superior Control of HIV-1 Replication by CD8+ T Cells Targeting Conserved Epitopes: Implications for HIV Vaccine Design
Source: PLoS One. 2013 May 31;8(5):e64405. doi: 10.1371/journal.pone.0064405 (PMC3669284; doi:10.1371/journal.pone.0064405)

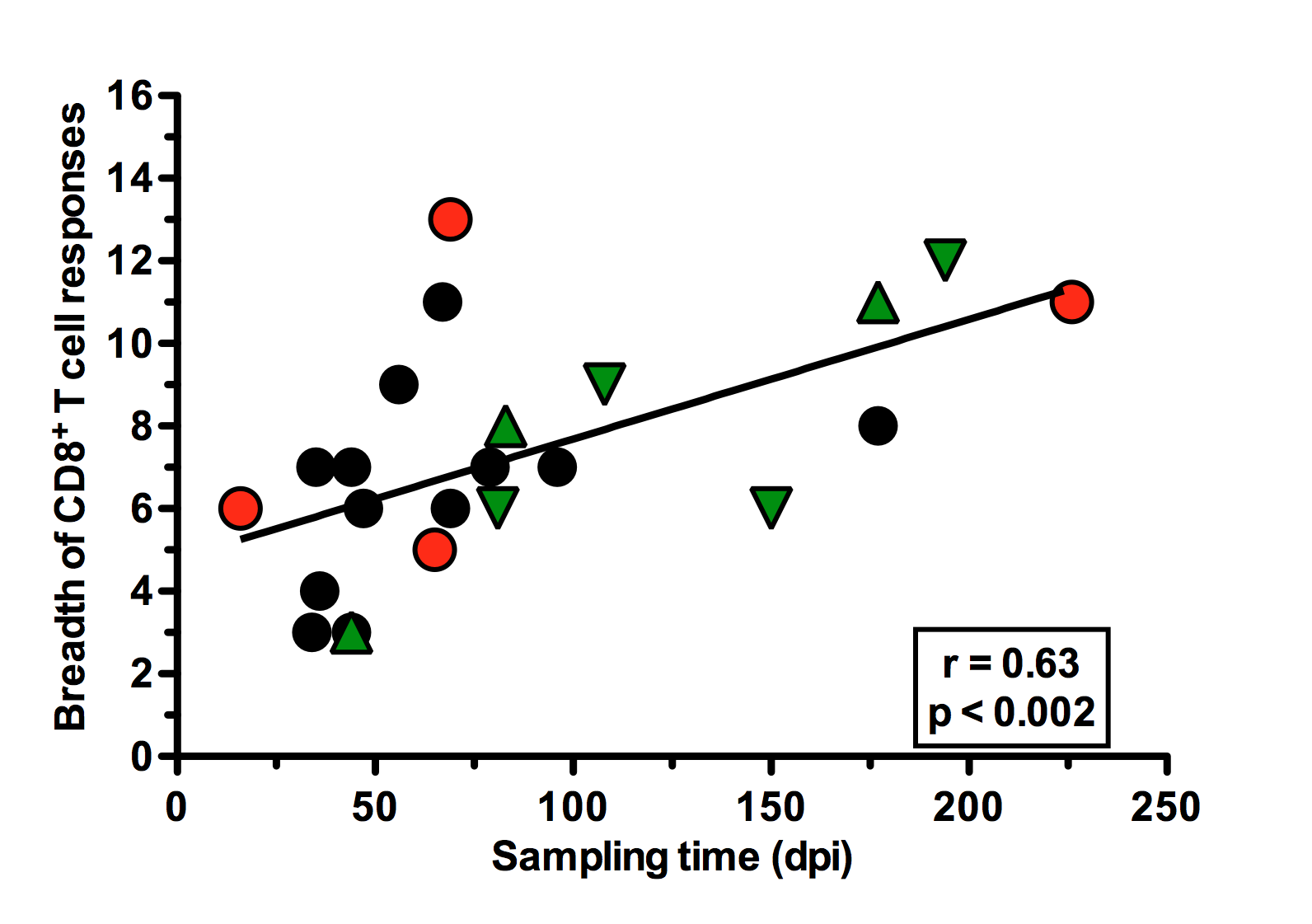

Supplement: Figure S1 — Assessment of HIV-1-specific CD8+ T cell responses during early HIV-1 infection. The number of HIV-1-specific CD8+ T cell epitopes was plotted for each subject against the sampling time, shown as days post infection (dpi). Subjects possessing B*35Px, B*27 and B*57 alleles are represented by red circles, green triangles and inverted green triangles respectively. (TIFF) [file pone.0064405.s001.tiff]

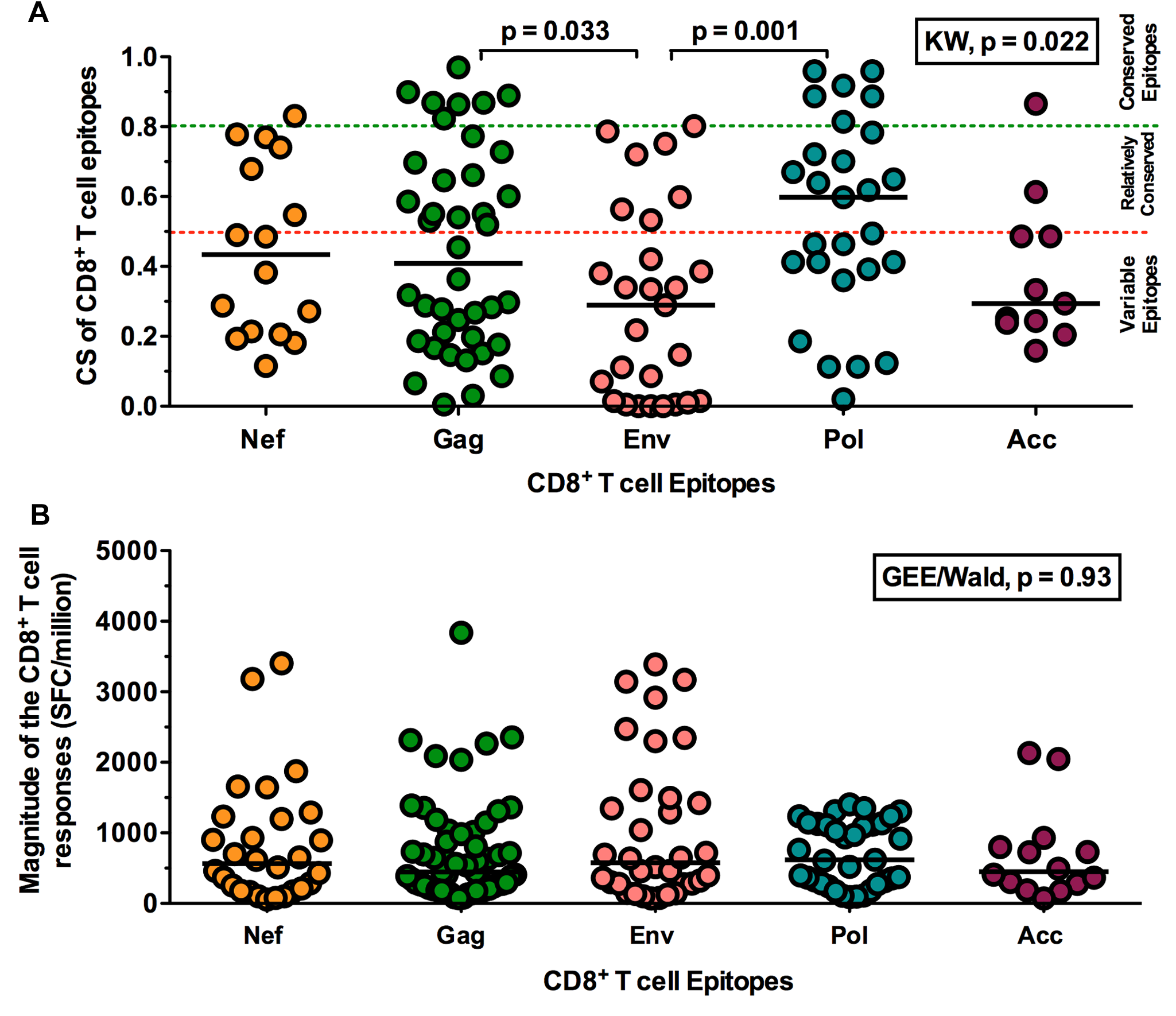

Supplement: Figure S2 — Majority of conserved epitopes (bCSp) targeted in early HIV-1 infections are Gag and Pol. The CS and magnitude of CD8+ T cell responses of total of 123 epitopes identified in 23 subjects were analyzed based on their protein types. (A) The median CS of CD8+ T cell epitopes by HIV protein types (Kruskal-Wallis, p = 0.022). (B) The median magnitude of responses (SFC/M) by protein types (Wald/GEE, p = 0.93). Horizontal lines indicate median. (TIFF) [file pone.0064405.s002.tiff]

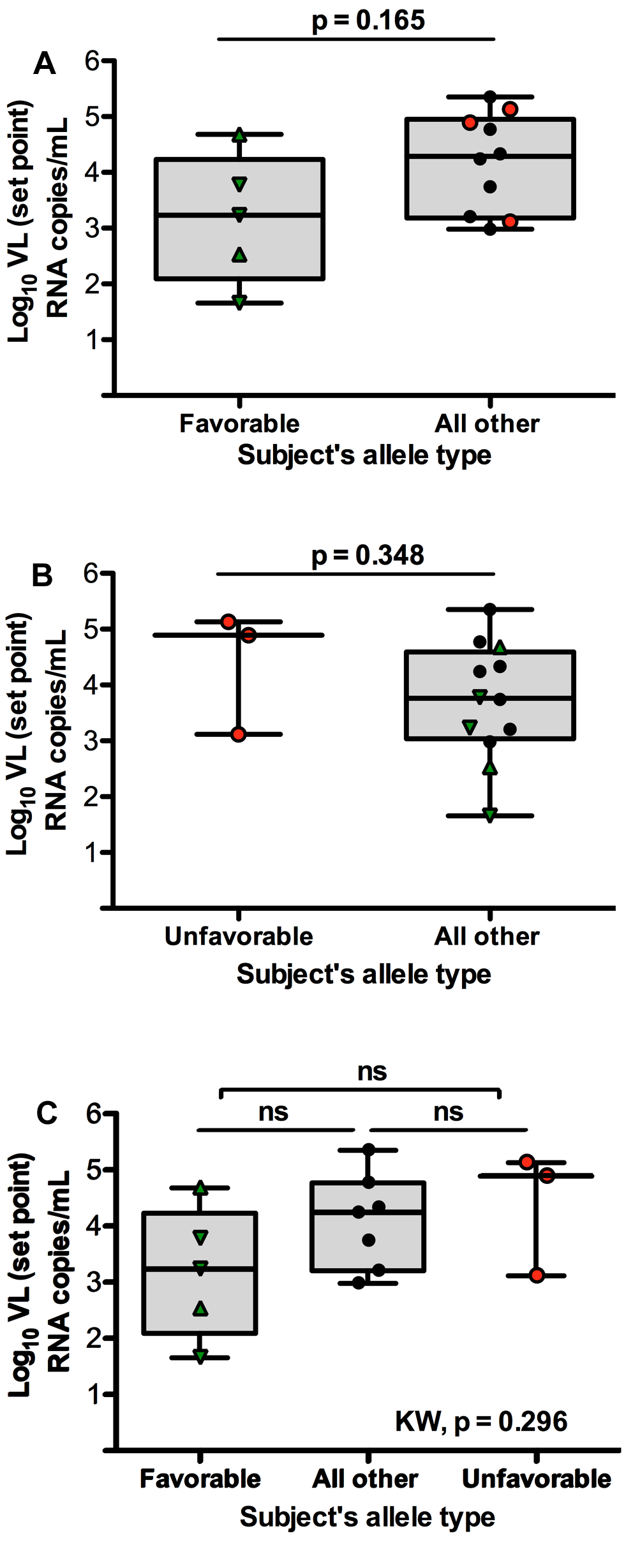

Supplement: Figure S3 — HIV-1 VL set points are not significantly different by HLA types. (A–C) The median plasma VL set point in individuals possessing favorable, unfavorable or all other alleles (Kruskal-Wallis, p = 0.296). Horizontal lines indicate median. Subjects possessing B*35Px, B*27 and B*57 alleles are represented by red circles, green triangles and inverted green triangles respectively (TIFF) [file pone.0064405.s003.tiff]

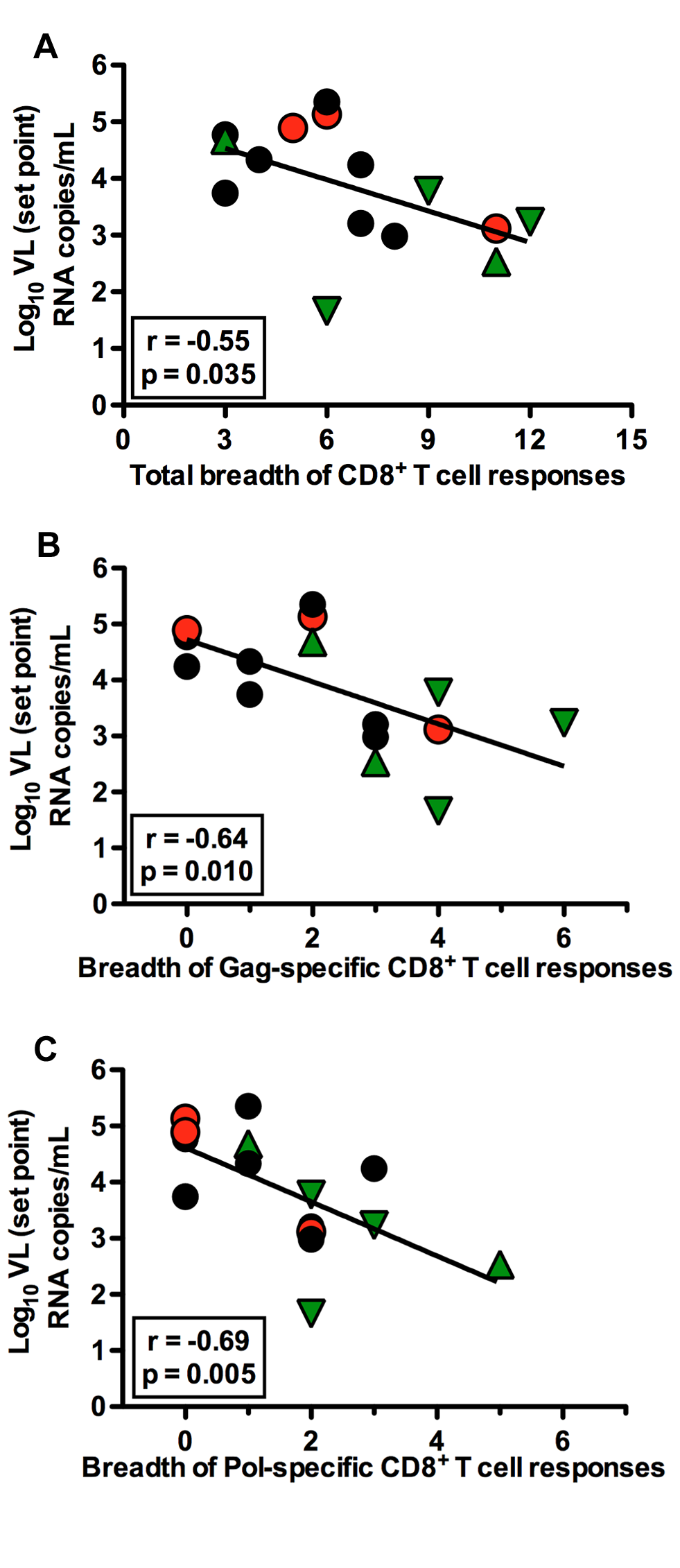

Supplement: Figure S4 — Correlation between breadth of HIV-1-specific CD8+ T cell responses and viremia. (A) Correlation between total breadth of CD8+ T cell responses and average plasma VL set point (Spearman Rank Correlation, r = −0.55, p = 0.035). (B and C) Correlation between breadth of CD8+ T cell responses against Gag or Pol epitopes with plasma VL set point (Spearman Rank Correlation, r = −0.64, p = 0.010 and r = −0.69, p = 0.005 respectively). (A–C) The solid line represents a regression line. Subject possessing B*35Px, B*27 and B*57 allele are represented by red circles, green triangles and inverted green triangles respectively. (TIFF) [file pone.0064405.s004.tiff]
