# Supplementary material for: Superior Control of HIV-1 Replication by CD8+ T Cells Targeting Conserved Epitopes: Implications for HIV Vaccine Design
Source: PLoS One. 2013 May 31;8(5):e64405. doi: 10.1371/journal.pone.0064405 (PMC3669284; doi:10.1371/journal.pone.0064405)
Supplement: Table S1 — HIV-1 specific CD8+ T cell responses in early infection: epitope specificity, MHC restriction, and frequency. (DOCX) [file pone.0064405.s005.docx]

| **Table S1. HIV-1 specific CD8^+^ T cell responses in early infection: epitope specificity, MHC restriction, and frequency** | | | | | | | | | |  |
| --- | --- | --- | --- | --- | --- | --- | --- | --- | --- | --- |
| **PTID.** | **HLA Class I type** | **HIV protein** | **HXB2 site^a^** | | **Epitope Sequence^b^** | **HLA restriction^c^** | | **SFC/10^6^ PBMC** | |  |
| 11439 | A*01:01, 29:02; B*38:01, 44:03; C*12:03, 16:01 | gp160 | 209 | 217 | SFEPIPIHY | A*2902 | | 1042 | |  |
|  |  | gp160 | 104 | 112 | MHEDIISLW | B*3801 | | 3390 | |  |
|  |  | gp160 | 376 | 384 | FNCGGEFFY | A29 | | 1288 | |  |
|  |  | gp160 | 620 | 628 | **NEIWDNMTW** | **B*4403** | | 3144 | |  |
|  |  | Protease | 34 | 42 | EEMNLPGRW | B*4403 | | 1406 | |  |
|  |  | Integrase | 10 | 19 | EEHEKYHSNW | B44*03 | | 760 | |  |
|  |  | Integrase | 227 | 235 | **YRDSRDPLW** | **B*3801** | | 1351 | |  |
| 53617 | A*02:01, 02:05; B*27:05, 49:01; C*02:02, 07:01 | Vpr | 31 | 39 | VRHFPRIWL | B27 | | 408 | |  |
|  |  | p17 | 11 | 19 | **GELDRWEKI** | **B*4901** | | 1033 | |  |
|  |  | p17 | 15 | 23 | **RWEKIRLRP** | **A*0205** | | 1013 | |  |
|  |  | p24 | 131 | 140 | KRWIILGLNK | B*2705 | | 1363 | |  |
|  |  | gp160 | 777 | 785 | **IVTRIVELL** | **A*0205** | | 278 | |  |
|  |  | gp160 | 786 | 794 | GRRGWEALK | B*2705 | | 103 | |  |
|  |  | Protease | 76 | 84 | LVGPTPVNI | A*0201 | | 181 | |  |
|  |  | Rnase | 60 | 68 | **QYALGIIQA** | **A*0201** | | 175 | |  |
|  |  | Rnase | 77 | 85 | **LVSQIIEQL** | **A*0205** | | 1086 | |  |
|  |  | Integrase | 186 | 194 | KRKGGIGGY | B*2705 | | 396 | |  |
|  |  | Integrase | 165 | 173 | **VRDQAEHLK** | A*0205 | | 606 | |  |
| 21746 | A*01:01; 48:01; B*40:01, 57:01; C*03:04, 06:02 | p24 | 30 | 40 | KAFSPEVIPMF | B*5701 | | 412 | |  |
|  |  | p24 | 15 | 23 | ISPRTLNAW | B*5701 | | 2355 | |  |
|  |  | p24 | 108 | 117 | TSTLQEQIGW | B*5701 | | 1356 | |  |
|  |  | p2p7p1p6 | 119 | 127 | **ELYPLASLR** |  | | 278 | |  |
|  |  | RT | 244 | 252 | IVLPEKDSW | B*5701 | | 1117 | |  |
|  |  | Integrase | 123 | 132 | STTVKAACWW | B57 | | 1138 | |  |
| 71101 | A*01:01; 03:01, B*08:01, 35:03; C*04:01, 07:01 | Nef | 68 | 76 | [FPVKPQVPL](http://www.hiv.lanl.gov/content/immunology/ctl_search?results=Search;id=54548) | B35 | | 1874 | |  |
|  |  | Nef | 90 | 97 | FLKEKGGL | B*0801 | | 255 | |  |
|  |  | p24 | 195 | 202 | NPDCKTIL | B*0801/B35 | | 203 | |  |
|  |  | p24 | 84 | 91 | HPVHAGPI | B35 | | 128 | |  |
|  |  | gp160 | 787 | 795 | RRGWEVLKY | A*0101 | | 2475 | |  |
|  |  | gp160 | 848 | 856 | RQGLERALL | B8/B*08 | | 136 | |  |
| 75688 | A*02:01, 32:01; B*08:01, 55:01; C*03:03, 07:01 | p24 | 128 | 135 | EIYKRWII | B*0801 | | 538 | |  |
|  |  | gp160 | 848 | 856 | RQGLERALL | B8 | | 2918 | |  |
|  |  | gp160 | 704 | 712 | **VINRVRQGY** |  | | 693 | |  |
| 41325 | A*29:02, 32:01; B*35G1, 44:03; C*04G1, 16:01 | Nef | 128 | 137 | TPGPGVRYPL | B35 | | 619 | |  |
|  |  | gp160 | 209 | 217 | SFEPIPIHY | A*2902 | | 274 | |  |
|  |  | gp160 | 704 | 712 | **VINRVRQGY** | B*4403 | | 77 | |  |
| 94153 | A*25:01, 68G1; B*14:02, 44G1; C*05:01, 08:02 | gp160 | 88 | 96 | NVTENFNMW | A*2501 | | 280 | |  |
|  |  | gp160 | 584 | 592 | ERYLRDQQL | B*14 | | 396 | |  |
|  |  | p24 | 13 | 23 | QAISPRTLNAW | A*2501 | | 220 | |  |
|  |  | p24 | 71 | 80 | ETINEEAAEW | A*2501 | | 447 | |  |
|  |  | p24 | 166 | 176 | DRFYKTLRAEQ | B*1402 | | 409 | |  |
|  |  | Protease | 34 | 42 | EEMNLPGRW | B44 | | 327 | |  |
|  |  | Integrase | 10 | 19 | EEHEKYHSNW | B*4403 | | 353 | |  |
| 20786 | A*02G1, 11:01; B*27:05, 40:02; C*02:02, 02:02 | p24 | 131 | 140 | KRWIILGLNK | B*2705 | | 1390 | |  |
|  |  | p2p7p1p6 | 64 | 71 | TERQANFL | B*4002 | | 270 | |  |
|  |  | Integrase | 186 | 194 | KRKGGIGGY | B*2705 | | 113 | |  |
| 17543 | A*01:01, 02:02; B*07G1, 15:03; C*02:10, 07:02 | Rev | 66 | 75 | RPAEPVPLQL | B7 | | 798 | |  |
|  |  | Nef | 183 | 191 | WRFDSRLAF | B*1503 | | 1656 | |  |
|  |  | p17 | 77 | 85 | SLYNTVATL | A*0202 | | 443 | |  |
|  |  | Integrase | 263 | 271 | RKAKIIRDY | B*1503 | | 1111 | |  |
| 25122 | A*01:01, 03G1; B*07G1, 37:01; C*06:02, 07:02 | Nef | 73 | 82 | QVPLRPMTYK | A*0301 | | 161 | |  |
|  |  | Nef | 105 | 115 | [KRQDILDLWVY](http://www.hiv.lanl.gov/content/immunology/ctl_search?results=Search;id=56248) | Cw7 | | 57 | |  |
|  |  | p17 | 71 | 79 | [GTEELRSLY](http://www.hiv.lanl.gov/content/immunology/ctl_search?results=Search;id=53820) | A*0101 | | 150 | |  |
|  |  | p17 | 87 | 95 | **CVHQRIEVK** | A*0301 | | 270 | |  |
|  |  | gp160 | 32 | 40 | **EKLWVTVYY** | Cw*0602,0702 | | 72 | |  |
|  |  | RT | 206 | 214 | **REHLLRWGF** | Cw*0702 | | 937 | |  |
| 53653 | A*24G1, 24G1; B*35:02, 38:01; C*04G1, 12:03 | Vif | 94 | 102 | **YSTQVDPDL** | B*3801 | | 725 | |  |
|  |  | Nef | 134 | 143 | RYPLTFGWCF | A*2402 | | 895 | |  |
|  |  | gp160 | 78 | 86 | DPNPQEVVL | B*3502 | | 460 | |  |
|  |  | gp160 | 44 | 52 | **VWKDAETTL** | B*3801 | | 458 | |  |
|  |  | gp160 | 621 | 629 | **EIWDNMTWL** | B*3801 | | 148 | |  |
| 51314 | A*03G1, 11:01; B*35:03, 53:01; C*04G1, 04G1 | gp160 | 621 | 629 | **DIWDNMTWM** | B*3503 | | 515 | |  |
|  |  | Nef | 73 | 82 | QVPLRPMTYK | A*0301 | | 285 | |  |
|  |  | Nef | 84 | 92 | AVDLSHFLK | A*0301/A*1101 | | 463 | |  |
|  |  | Nef | 135 | 143 | YPLTFGWCF | B*5301 | | 169 | |  |
|  |  | Nef | 175 | 183 | **DPEKEVLVW** | B*5301 | | 366 | |  |
|  |  | p17 | 20 | 28 | RLRPGGKKK | A*0301 | | 487 | |  |
|  |  | p17 | 83 | 91 | ATLYCVHQR | A*1101 | | 1148 | |  |
|  |  | p24 | 195 | 202 | NPDCKTIL | B*3503 | | 87 | |  |
|  |  | p24 | 176 | 184 | QASQDVKNW | B*5301 | | 183 | |  |
|  |  | RT | 269 | 277 | QIYAGIKVK | A*03/A*1101 | | 243 | |  |
|  |  | RT | 520 | 528 | QIIEQLIKK | A*1101 | | 88 | |  |
| 44149 | A*01:01, 24G1; B*07G1, 08G1; C*07G1, 07:02 | gp160 | 843 | 851 | IPRRIRQGL | B*0702 | | 717 | |  |
|  |  | p17 | 93 | 101 | DIKDTKEAL | B*0801 | | 2316 | |  |
|  |  | p24 | 195 | 202 | NPDCKTIL | B*0801 | | 83 | |  |
|  |  | p24 | 128 | 135 | EIYKRWII | B*0801 | | 381 | |  |
|  |  | Nef | 90 | 97 | FLKEKGGL | B*0801 | | 100 | |  |
|  |  | Nef | 134 | 143 | RYPLTFGWCF | A*2402 | | 510 | |  |
|  |  | RT | 156 | 164 | SPAIFQSSM | B7 | | 917 | |  |
|  |  | RT | 18 | 26 | GPKVKQWPL | B*0801 | | 97 | |  |
| 10849 | A*23:01, 68G1; B*3543, 57:03; C*01:02, 02:10 | Vif | 61 | 69 | **DARLVITTY** | B*3501 | | 277 | |  |
|  |  | gp160 | 217 | 226 | YCAPAGFAIL | Cw*0102 | | 322 | |  |
|  |  | p24 | 15 | 23 | ISPRTLNAW | B*5701 | | 644 | |  |
|  |  | p24 | 145 | 153 | YSPVSILDI | Cw*0102 | | 252 | |  |
|  |  | p24 | 30 | 40 | KAFSPEVIPMF | B*5701 | | 697 | |  |
|  |  | p24 | 108 | 117 | TSTLQEQIAW | B*5703 | | 812 | |  |
|  |  | Nef | 116 | 124 | HTQGYFPDW | B57 | | 176 | |  |
|  |  | Pol | 23 | 31 | **ANSPTSREL** |  | | 286 | |  |
|  |  | Integrase | 95 | 104 | QETAYFILKL | A*6802 | | 619 | |  |
| 67200 | A*01:01, 24G1; B*08:01, 57:01; C*06:02, 07G1 | gp160 | 805 | 814 | **QELKNSAVSL** | B*0801 | | 143 | |  |
|  |  | p17 | 71 | 79 | GSEELRSLY | A*0101/B*57 | | 983 | |  |
|  |  | p17 | 87 | 95 | **CVHQRIEVK** | A*02 | | 633 | |  |
|  |  | p24 | 128 | 136 | EIYKRWIIL | B*0801 | | 191 | |  |
|  |  | p24 | 30 | 40 | KAFSPEVIPMF | B*5701 | | 716 | |  |
|  |  | p24 | 108 | 117 | TSTLQEQIAW | B*57 | | 239 | |  |
|  |  | p24 | 176 | 184 | QASQEVKNW | B*5701 | | 138 | |  |
|  |  | Nef | 90 | 97 | FLKEKGGL | B*0801 | | 1198 | |  |
|  |  | Nef | 116 | 124 | HTQGYFPDW | B*5701 | | 212 | |  |
|  |  | RT | 127 | 135 | YTAFTIPSV | A2 | | 1235 | |  |
|  |  | RT | 244 | 252 | IVLPEKDSW | B*5701 | | 1306 | |  |
|  |  | RT | 375 | 383 | ITTESIVIW | B*5701 | | 318 | |  |
| 25327 | A*23:01, 26:01; B*38:01, 38:01; C*04G1, 12:03 | Vif | 79 | 87 | [WHLGQGVSI](http://www.hiv.lanl.gov/content/immunology/ctl_search?results=Search;id=52563) | B*3801 | | 2132 | |  |
|  |  | gp160 | 104 | 112 | MHEDIISLW | B*3801 | | 1497 | |  |
|  |  | gp160 | 53 | 61 | **FCASDAKSY** | **B*3801** | | 650 | |  |
|  |  | Integrase | 11 | 19 | **EHEKYHNNW** | **B*3801** | | 973 | |  |
|  |  | Integrase | 95 | 103 | **QETAYFILK** |  | | 375 | |  |
|  |  | Integrase | 227 | 235 | **YRDSRDPLW** | **B*3801** | | 1020 | |  |
| 63794 | A*02G1, 02G1; B*07G1, 44G1; C*07:02, 07:04 | Nef | 77 | 85 | RPMTYKAAV |  | | 698 | |  |
|  |  | Nef | 68 | 76 | [FPVKPQVPL](http://www.hiv.lanl.gov/content/immunology/ctl_search?results=Search;id=54548) | B7 | | 1648 | |  |
|  |  | p2p7p1p6 | 70 | 77 | FLGKIWPS | A*0201 | | 388 | |  |
|  |  | p24 | 223 | 231 | GPSHKARVL | B*0702 | | 2035 | |  |
|  |  | gp160 | 200 | 208 | **AITQACPKV** | **A*02G1** | | 1608 | |  |
|  |  | gp160 | 298 | 307 | RPNNNTRKSI | B*0702 | | 303 | |  |
|  |  | gp160 | 843 | 851 | IPRRIRQGL | B*0702 | | 3170 | |  |
|  |  | gp160 | 700 | 708 | AVLSVVNRV | A2 | | 125 | |  |
|  |  | RT | 156 | 164 | SPAIFQSSM | B7 | | 1310 | |  |
| 79379 | A*01:01, 01:01; B*27:05, 52:01; C*02:01, 12:02 | Tat | 39 | 47 | **ITKGLGISY** |  | | 305 | |  |
|  |  | Vpr | 31 | 39 | VRHFPRIWL | B27 | | 73 | |  |
|  |  | gp160 | 836 | 845 | **IGRAILHIPR** | B*2705 | | 638 | |  |
|  |  | p17 | 19 | 27 | IRLRPGGKK | B*2705 | | 145 | |  |
|  |  | p17 | 71 | 79 | [GTEELRSLY](http://www.hiv.lanl.gov/content/immunology/ctl_search?results=Search;id=53820) | A*0101 | | 1308 | |  |
|  |  | p17 | 81 | 89 | **TVATLYCVH** |  | | 345 | |  |
|  |  | p24 | 131 | 140 | KRWIILGLNK | B*2705 | | 2265 | |  |
|  |  | Integrase | 186 | 194 | KRKGGIGGY | B*2705 | | 1238 | |  |
| 57604 | A*02G1, 02G1; B*13:02, 35G1; C*04:19, 06:02 | Vif | 61 | 69 | **DARLVITTY** | B*35 | | 173 | |  |
|  |  | Nef | 74 | 81 | VPLRPMTY | B*3501 | | 430 | |  |
|  |  | Nef | 128 | 137 | TPGPGVRYPL | B35 | | 1233 | |  |
|  |  | Nef | 134 | 143 | RYPLTFGWCF | B*35 | | 80 | |  |
|  |  | p17 | 77 | 85 | SLYNTVATL | A*0201 | | 683 | |  |
|  |  | p24 | 122 | 130 | PPIPVGDIY | B*3501 | | 210 | |  |
|  |  | p24 | 8 | 17 | **GQMVHQAISP** |  | | 2090 | |  |
| 98621 | A*02G1, 02G1; B*40G1, 44G1; C*03:04, 05:01 | Vif | 102 | 111 | **LADQLIHLYY** |  | | 493 | |  |
|  |  | Vpr | 12 | 20 | **REPYNEWTL** | B*4002 | | 731 | |  |
|  |  | Nef | 92 | 100 | KEKGGLEGL | B*4001 | | 1290 | |  |
|  |  | Nef | 83 | 91 | AAVDLSHFL | A*02 | | 100 | |  |
|  |  | p17 | 119 | 128 | **AADTGNSSQV** |  | | 1186 | |  |
|  |  | p24 | 174 | 184 | AEQASQDVKNW | B*4402 | | 3836 | |  |
|  |  | p24 | 118 | 126 | MTSNPPIPV | A*0201 | | 178 | |  |
|  |  | p2p7p1p6 | 70 | 77 | FLGKIWPS | A*201 | | 561 | |  |
|  |  | p6 | 33 | 41 | KELYPLASL | B*40 | | 298 | |  |
|  |  | RT | 181 | 189 | YQYMDDLYV | A*0201 | | 518 | |  |
|  |  | gp160 | 805 | 814 | QELKNSAVSL | B*4001 | | 1348 | |  |
| 32645 | A*01:01, 02G1; B*08:01, 35:03; C*04G1, 07G1 | Vif | 101 | 109 | GLADQLIHL | | A*0201 | | 2047 | |
|  |  | Vif | 149 | 157 | ALAALITPK | | A2 | | 929 | |
|  |  | Nef | 90 | 97 | FLKEKGGL | | B*0801 | | 3181 | |
|  |  | p17 | 77 | 85 | SLYNTVATL | | A*0201 | | 194 | |
|  |  | p17 | 74 | 82 | ELRSLYNTV | | B*0801 | | 626 | |
|  |  | p24 | 128 | 135 | EIYKRWII | | B*0801 | | 184 | |
|  |  | p2 | 6 | 14 | **QVTNSATIM** | | B*3503 | | 76 | |
|  |  | RT | 107 | 115 | TVLDVGDAY | | B*35 | | 1129 | |
|  |  | Rnase | 21 | 29 | **RGRQKVVSL** | | B*0801 | | 111 | |
|  |  | gp160 | 78 | 86 | DPNPQEVVL | | B*35 | | 2346 | |
|  |  | gp160 | 606 | 614 | TAVPWNASW | | B*35 | | 2300 | |
|  |  | gp160 | 848 | 856 | **RQGFERALL** | | A*02 | | 1423 | |
|  |  | gp160 | 814 | 822 | **LLNTTAIVV** | A*0201 | | 362 | |  |
| 51729 | A*24G1, 24G1; B*35:05, 40G1; C*03:04, 04G1 | Vif | 116 | 124 | **SESAIRNAI** | B*4002 | | 367 | |  |
|  |  | Vpr | 12 | 20 | **REPYNEWTL** | B*4002 | | 187 | |  |
|  |  | Nef | 92 | 100 | KEKGGLEGL | B*4001 | | 3405 | |  |
|  |  | Nef | 134 | 143 | RYPLTFGWCF | A*2402 | | 904 | |  |
|  |  | p17 | 28 | 36 | KYKLKHIVW | A*2402 | | 601 | |  |
|  |  | p17 | 92 | 101 | IEIKDTKEAL | B*4001 | | 293 | |  |
|  |  | p6 | 33 | 41 | KELYPLASL | B*40 | | 735 | |  |
| 44091 | A*02G1, 24G1; B*44G1, 57:01; C*05:01, 60:02 | Nef | 134 | 141 | RYPLTFGW | A*2402 | | 929 | |  |
|  |  | Nef | 116 | 124 | HTQGYFPDW | B57 | | 654 | |  |
|  |  | p24 | 108 | 117 | TSTLQEQIGW | B*5701 | | 874 | |  |
|  |  | RT | 244 | 252 | IVLPEKDSW | B*5701 | | 366 | |  |
|  |  | Integrase | 114 | 123 | **HTDNGSNFTS** |  | | 1146 | |  |
|  |  | Integrase | 838 | 846 | STTVKAACW | B*5701 | | 274 | |  |

**^a^** Epitope position (based on HXB2 amino acid sequence) in HIV-1 proteins

**^b^** Amino acid sequence of identified new T-cell epitopes, with newly defined HLA restriction shown in bold

**^c^** Predicted restricting HLA allele of the novel epitope, with the newly confirmed HLA restriction shown in bold
