# Supplementary material for: Superior Control of HIV-1 Replication by CD8+ T Cells Targeting Conserved Epitopes: Implications for HIV Vaccine Design
Source: PLoS One. 2013 May 31;8(5):e64405. doi: 10.1371/journal.pone.0064405 (PMC3669284; doi:10.1371/journal.pone.0064405)
Supplement: Table S2 — HIV-1 specific CD8+ T cell responses in early infection: comparison of CD8+ T cell epitope-conservation by different methods. (DOCX) [file pone.0064405.s006.docx]

| **Table S2. HIV-1 specific CD8^+^ T cell responses in early infection: comparison of CD8^+^ T cell epitope-conservation by different methods.** | | | | | | | | | |
| --- | --- | --- | --- | --- | --- | --- | --- | --- | --- |
|  |  |  |  |  |  |  |  |  |  |
| **Epitope Sequence^a^** | **HXB2 site^b^** | | **HIV protein** | **bCSp^c^** | **mCSp^d^** | **bCSe^e^** | **mCSe^f^** | **bCSconseq^g^** | **mCSconseq^h^** |
| VINRVRQGY | 704 | 712 | Env | 0 | 0.042 | 0.342 | 0.437 | -0.463 | -0.368 |
| EIWDNMTWL | 621 | 629 | Env | 0 | 0.049 | 0.543 | 0.632 | 0.086 | 0.506 |
| VWKDAETTL | 44 | 52 | Env | 0 | 0.077 | 0.149 | 0.432 | -0.465 | -0.443 |
| FCASDAKSY | 53 | 61 | Env | 0.005 | 0.086 | 0.096 | 0.156 | -0.503 | -0.617 |
| AITQACPKV | 200 | 208 | Env | 0.005 | 0.262 | 0.205 | 0.353 | -0.384 | -0.498 |
| IGRAILHIPR | 836 | 845 | Env | 0.015 | 0.023 | 0.648 | 0.729 | 0.022 | 0.308 |
| LLNTTAIVV | 814 | 822 | Env | 0.015 | 0.061 | 0.572 | 0.726 | -0.135 | 0.559 |
| NEIWDNMTW | 620 | 628 | Env | 0.071 | 0.04 | 0.69 | 0.729 | 0.52 | 0.925 |
| DIWDNMTWM | 621 | 629 | Env | 0.086 | 0.09 | 0.543 | 0.632 | 0.086 | 0.506 |
| RQGFERALL | 848 | 856 | Env | 0.112 | 0.15 | 0.456 | 0.563 | -0.306 | 0.249 |
| EKLWVTVYY | 32 | 40 | Env | 0.218 | 0.073 | 0.313 | 0.386 | 0.014 | -0.339 |
| IVTRIVELL | 777 | 785 | Env | 0.335 | 0.101 | 0.575 | 0.792 | -0.213 | 0.206 |
| QELKNSAVSL | 805 | 814 | ENV | 0.563 | 0.157 | 0.452 | 0.644 | -0.381 | -0.18 |
| AVLSVVNRV | 700 | 708 | Env | 0.01 | 0.025 | 0.511 | 0.845 | -0.231 | -0.345 |
| RRGWEVLKY | 787 | 795 | Env | 0.147 | 0.049 | 0.479 | 0.984 | -0.062 | -0.17 |
| TAVPWNASW | 606 | 614 | Env | 0.289 | 0.075 | 0.375 | 0.458 | -0.242 | -0.245 |
| IPRRIRQGL | 843 | 851 | Env | 0.34 | 0.296 | 0.471 | 0.485 | -0.234 | 0.192 |
| ERYLRDQQL | 584 | 592 | Env | 0.34 | 0.176 | 0.222 | 0.351 | -0.407 | -0.343 |
| DPNPQEVVL | 78 | 86 | Env | 0.381 | 0.118 | 0.354 | 0.517 | -0.18 | -0.126 |
| GRRGWEALK | 786 | 794 | Env | 0.386 | 0.153 | 0.479 | 0.667 | -0.063 | 0.036 |
| RPNNNTRKSI | 298 | 307 | Env | 0.421 | 0.277 | 0.441 | 0.599 | -0.139 | -0.299 |
| RQGLERALL | 848 | 856 | Env | 0.533 | 0.33 | 0.456 | 0.563 | -0.306 | 0.249 |
| YCAPAGFAIL | 217 | 226 | Env | 0.599 | 0.464 | 0.182 | 0.196 | -0.369 | -0.589 |
| NVTENFNMW | 88 | 96 | Env | 0.721 | 0.66 | 0.166 | 0.18 | -0.424 | -0.589 |
| SFEPIPIHY | 209 | 217 | Env | 0.751 | 0.352 | 0.122 | 0.253 | -0.464 | -0.544 |
| MHEDIISLW | 104 | 112 | Env | 0.787 | 0.525 | 0.124 | 0.234 | -0.486 | -0.589 |
| FNCGGEFFY | 376 | 384 | Env | 0.802 | 0.512 | 0.147 | 0.226 | -0.441 | -0.595 |
| CVHQRIEVK | 87 | 95 | Gag | 0.152 | 0.094 | 0.359 | 0.674 | 0.688 | 0.908 |
| QVTNSATIM | 6 | 14 | Gag | 0.187 | 0.063 | 0.964 | 1.17 | 2.008 | 2.149 |
| AADTGNSSQV | 119 | 128 | Gag | 0.212 | 0.078 | 0.856 | 1.463 | 1.689 | 1.898 |
| ELYPLASLR | 119 | 127 | Gag | 0.247 | 0.079 | 0.514 | 0.702 | 0.868 | 1.161 |
| GELDRWEKI | 11 | 19 | Gag | 0.364 | 0.132 | 0.279 | 0.392 | 0.473 | 0.264 |
| RWEKIRLRP | 15 | 23 | Gag | 0.53 | 0.218 | 0.169 | 0.282 | 0.079 | 0.061 |
| GQMVHQAISP | 8 | 17 | Gag | 0.551 | 0.422 | 0.155 | 0.28 | -0.034 | 0.209 |
| TVATLYCVH | 81 | 89 | Gag | 0.551 | 0.604 | 0.184 | 0.253 | 0.1 | -0.012 |
| GTEELRSLY | 71 | 79 | Gag | 0.005 | 0.121 | 0.251 | 0.351 | 0.505 | 0.39 |
| DIKDTKEAL | 93 | 101 | Gag | 0.03 | 0.023 | 0.214 | 0.328 | 0.26 | 0.173 |
| TSTLQEQIAW | 108 | 117 | Gag | 0.066 | 0.244 | 0.154 | 0.253 | -0.118 | -0.016 |
| PPIPVGDIY | 122 | 130 | Gag | 0.086 | 0.241 | 0.109 | 0.187 | -0.323 | -0.222 |
| GPSHKARVL | 223 | 231 | Gag | 0.131 | 0.381 | 0.153 | 0.158 | -0.045 | -0.134 |
| MTSNPPIPV | 118 | 126 | Gag | 0.146 | 0.3 | 0.204 | 0.263 | -0.038 | -0.023 |
| IEIKDTKEAL | 92 | 101 | Gag | 0.167 | 0.103 | 0.201 | 0.32 | 0.189 | 0.128 |
| YSPVSILDI | 145 | 153 | Gag | 0.177 | 0.601 | 0.185 | 0.161 | -0.105 | -0.427 |
| ELRSLYNTV | 74 | 82 | Gag | 0.197 | 0.192 | 0.322 | 0.34 | 0.736 | 0.533 |
| KYKLKHIVW | 28 | 36 | Gag | 0.268 | 0.12 | 0.305 | 0.485 | 0.428 | 0.77 |
| AEQASQDVKNW | 174 | 184 | Gag | 0.278 | 0.162 | 0.164 | 0.23 | -0.08 | -0.193 |
| QASQDVKNW | 176 | 184 | Gag | 0.283 | 0.163 | 0.187 | 0.274 | 0.027 | -0.08 |
| GSEELRSLY | 71 | 79 | Gag | 0.288 | 0.124 | 0.251 | 0.351 | 0.505 | 0.39 |
| ATLYCVHQR | 83 | 91 | Gag | 0.298 | 0.189 | 0.258 | 0.495 | 0.316 | 0.482 |
| SLYNTVATL | 77 | 85 | Gag | 0.318 | 0.326 | 0.286 | 0.287 | 0.57 | 0.25 |
| KELYPLASL | 33 | 41 | Gag | 0.455 | 0.174 | 0.462 | 0.748 | 0.588 | 1.158 |
| QASQEVKNW | 176 | 184 | Gag | 0.52 | 0.183 | 0.187 | 0.274 | 0.027 | -0.08 |
| QAISPRTLNAW | 13 | 23 | Gag | 0.54 | 0.425 | 0.15 | 0.208 | -0.03 | 0.056 |
| HPVHAGPI | 84 | 91 | Gag | 0.586 | 0.501 | 0.261 | 0.296 | 0.225 | 0.179 |
| TERQANFL | 64 | 71 | Gag | 0.601 | 0.792 | 0.25 | 0.193 | -0.251 | -0.44 |
| RLRPGGKKK | 20 | 28 | Gag | 0.646 | 0.446 | 0.16 | 0.253 | 0.013 | 0.039 |
| FLGKIWPS | 70 | 77 | Gag | 0.662 | 0.753 | 0.17 | 0.19 | -0.442 | -0.371 |
| ISPRTLNAW | 15 | 23 | Gag | 0.697 | 0.593 | 0.115 | 0.134 | -0.129 | -0.188 |
| TSTLQEQIGW | 108 | 117 | Gag | 0.727 | 0.397 | 0.154 | 0.253 | -0.118 | -0.016 |
| EIYKRWIIL | 128 | 136 | Gag | 0.773 | 0.528 | 0.137 | 0.165 | -0.142 | -0.261 |
| KRWIILGLNK | 131 | 140 | Gag | 0.823 | 0.866 | 0.108 | 0.092 | -0.29 | -0.487 |
| EIYKRWII | 128 | 135 | Gag | 0.864 | 0.559 | 0.099 | 0.155 | -0.345 | -0.33 |
| IRLRPGGKK | 19 | 27 | Gag | 0.869 | 0.822 | 0.063 | 0.102 | -0.37 | -0.348 |
| ETINEEAAEW | 71 | 80 | Gag | 0.869 | 0.372 | 0.065 | 0.137 | -0.413 | -0.45 |
| NPDCKTIL | 195 | 202 | Gag | 0.889 | 0.787 | 0.095 | 0.134 | -0.419 | -0.489 |
| DRFYKTLRAEQ | 166 | 176 | Gag | 0.899 | 0.396 | 0.069 | 0.143 | -0.51 | -0.515 |
| KAFSPEVIPMF | 30 | 40 | Gag | 0.97 | 0.84 | 0.027 | 0.096 | -0.536 | -0.514 |
| DPEKEVLVW | 175 | 183 | Nef | 0.181 | 0.036 | 0.347 | 0.659 | 0.176 | 0.648 |
| FPVKPQVPL | 68 | 76 | Nef | 0.115 | 0.086 | 0.126 | 0.143 | -0.39 | -0.507 |
| TPGPGVRYPL | 128 | 137 | Nef | 0.193 | 0.338 | 0.269 | 0.233 | 0.198 | -0.214 |
| AAVDLSHFL | 83 | 91 | Nef | 0.206 | 0.089 | 0.304 | 0.408 | 0.254 | 0.13 |
| RPMTYKAAV | 77 | 85 | Nef | 0.214 | 0.105 | 0.32 | 0.238 | 0.352 | -0.069 |
| WRFDSRLAF | 183 | 191 | Nef | 0.272 | 0.067 | 0.245 | 0.462 | -0.024 | -0.222 |
| AVDLSHFLK | 84 | 92 | Nef | 0.288 | 0.141 | 0.26 | 0.326 | -0.02 | -0.217 |
| KRQDILDLWVY | 105 | 115 | Nef | 0.383 | 0.224 | 0.207 | 0.227 | -0.158 | -0.325 |
| RYPLTFGWCF | 134 | 143 | Nef | 0.486 | 0.427 | 0.183 | 0.22 | -0.127 | -0.165 |
| YPLTFGWCF | 135 | 143 | Nef | 0.49 | 0.431 | 0.186 | 0.239 | -0.091 | -0.098 |
| RYPLTFGW | 134 | 141 | Nef | 0.547 | 0.582 | 0.201 | 0.187 | -0.041 | -0.254 |
| KEKGGLEGL | 92 | 100 | Nef | 0.679 | 0.497 | 0.159 | 0.167 | -0.324 | -0.513 |
| HTQGYFPDW | 116 | 124 | Nef | 0.741 | 0.359 | 0.125 | 0.185 | -0.388 | -0.239 |
| QVPLRPMTYK | 73 | 82 | Nef | 0.77 | 0.719 | 0.098 | 0.143 | -0.49 | -0.456 |
| FLKEKGGL | 90 | 97 | Nef | 0.778 | 0.908 | 0.09 | 0.077 | -0.491 | -0.715 |
| VPLRPMTY | 74 | 81 | Nef | 0.831 | 0.744 | 0.085 | 0.147 | -0.529 | -0.412 |
| REHLLRWGF | 206 | 214 | Pol | 0.021 | 0.135 | 0.226 | 0.392 | 0.659 | 0.773 |
| YTAFTIPSV | 127 | 135 | Pol | 0.113 | 0.122 | 0.093 | 0.148 | -0.047 | -0.124 |
| ANSPTSREL | 23 | 31 | Pol | 0.113 | 0.307 | 0.335 | 0.459 | 0.925 | 0.755 |
| EHEKYHNNW | 11 | 19 | Pol | 0.124 | 0.176 | 0.212 | 0.35 | 0.154 | 0.363 |
| RGRQKVVSL | 21 | 29 | Pol | 0.392 | 0.298 | 0.32 | 0.474 | 0.273 | 0.548 |
| QETAYFILK | 95 | 103 | Pol | 0.412 | 0.377 | 0.192 | 0.316 | 0.116 | 0.226 |
| LVSQIIEQL | 77 | 85 | Pol | 0.598 | 0.189 | 0.249 | 0.405 | 0.44 | 0.421 |
| HTDNGSNFTS | 114 | 123 | pol | 0.639 | 0.772 | 0.209 | 0.221 | -0.045 | -0.34 |
| YRDSRDPLW | 227 | 235 | Pol | 0.649 | 0.153 | 0.295 | 0.308 | 0.477 | -0.142 |
| QYALGIIQA | 60 | 68 | Pol | 0.887 | 0.898 | 0.114 | 0.154 | -0.325 | -0.442 |
| VRDQAEHLK | 165 | 173 | Pol | 0.887 | 0.62 | 0.158 | 0.269 | -0.376 | -0.149 |
| ITTESIVIW | 375 | 383 | Pol | 0.186 | 0.063 | 0.249 | 0.494 | 0.561 | 0.626 |
| QIYAGIKVK | 269 | 277 | Pol | 0.361 | 0.218 | 0.191 | 0.315 | 0.508 | 0.657 |
| EEMNLPGRW | 34 | 42 | Pol | 0.412 | 0.072 | 0.278 | 0.374 | 0.73 | 0.759 |
| QETAYFILKL | 95 | 104 | Pol | 0.412 | 0.377 | 0.18 | 0.298 | 0.054 | 0.149 |
| STTVKAACWW | 123 | 132 | Pol | 0.464 | 0.088 | 0.251 | 0.334 | 0.245 | 0.09 |
| STTVKAACW | 838 | 846 | Pol | 0.464 | 0.072 | 0.266 | 0.356 | 0.319 | 0.164 |
| EEHEKYHSNW | 10 | 19 | Pol | 0.495 | 0.26 | 0.232 | 0.352 | 0.29 | 0.383 |
| IVLPEKDSW | 244 | 252 | Pol | 0.619 | 0.143 | 0.151 | 0.428 | 0.138 | 0.691 |
| SPAIFQSSM | 156 | 164 | Pol | 0.67 | 0.649 | 0.117 | 0.162 | -0.155 | -0.247 |
| RKAKIIRDY | 263 | 271 | Pol | 0.701 | 0.537 | 0.208 | 0.322 | 0.05 | 0.141 |
| QIIEQLIKK | 520 | 528 | Pol | 0.722 | 0.444 | 0.192 | 0.401 | 0.091 | 0.207 |
| LVGPTPVNI | 76 | 84 | Pol | 0.784 | 0.829 | 0.09 | 0.085 | -0.127 | -0.348 |
| KRKGGIGGY | 186 | 194 | Pol | 0.814 | 0.862 | 0.2 | 0.202 | -0.011 | -0.322 |
| YQYMDDLYV | 181 | 189 | Pol | 0.918 | 0.945 | 0.052 | 0.056 | -0.308 | -0.477 |
| TVLDVGDAY | 107 | 115 | Pol | 0.959 | 0.951 | 0.03 | 0.044 | -0.444 | -0.526 |
| GPKVKQWPL | 18 | 26 | Pol | 0.959 | 0.865 | 0.02 | 0.069 | -0.431 | -0.347 |
| RPAEPVPLQL | 66 | 75 | Rev | 0.251 | 0.157 | 0.394 | 0.436 | 0.242 | 0.211 |
| ITKGLGISY | 39 | 47 | Tat | 0.244 | 0.116 | 0.352 | 0.405 | 0.026 | -0.356 |
| DARLVITTY | 61 | 69 | Vif | 0.159 | 0.068 | 0.467 | 0.615 | 0.418 | 0.53 |
| YSTQVDPDL | 94 | 102 | Vif | 0.239 | 0.114 | 0.434 | 0.478 | 0.212 | -0.071 |
| SESAIRNAI | 116 | 124 | Vif | 0.294 | 0.116 | 0.463 | 0.5 | 0.199 | 0.019 |
| LADQLIHLYY | 102 | 111 | Vif | 0.486 | 0.209 | 0.325 | 0.408 | -0.04 | -0.087 |
| ALAALITPK | 149 | 157 | Vif | 0.205 | 0.072 | 0.476 | 0.628 | 0.254 | 0.162 |
| GLADQLIHL | 101 | 109 | Vif | 0.486 | 0.059 | 0.292 | 0.362 | -0.155 | -0.224 |
| WHLGQGVSI | 79 | 87 | Vif | 0.865 | 0.471 | 0.172 | 0.256 | -0.486 | -0.513 |
| REPYNEWTL | 12 | 20 | Vpr | 0.613 | 0.497 | 0.209 | 0.302 | 0.024 | 0.004 |
| VRHFPRIWL | 31 | 39 | Vpr | 0.333 | 0.121 | 0.289 | 0.277 | 0.148 | -0.005 |

^a^ Amino acid sequence of identified new T-cell epitopes, with newly defined HLA restriction shown in bold

^b^ Epitope position (based on HXB2 amino acid sequence) in HIV-1 proteins

_c_ bCSp is defined as the frequency of the exact epitope matches in aligned clade-B sequences

^d^ mCSp is defined as the frequency of the exact epitope matches in aligned group-M sequences

^e^ bCSe is defined as the average entropy score of all the positions along the epitope based on aligned clade-B sequences.

^f^ mCSe is defined as the average entropy score of all the positions along the epitope based on aligned group-M sequences

^g^ bCSconseq is defined by averaging conseq scores computed from a phylogenetic tree built over a set of aligned group-B sequences.

^h^ bCSconseq is defined by averaging conseq scores computed from a phylogenetic tree built over a set of aligned group-M sequences.
